# Cherry-Net: real-time segmentation algorithm of cherry maturity based on improved PIDNet

**DOI:** 10.3389/fpls.2025.1607205

**Published:** 2025-09-03

**Authors:** Jie Cui, Lilian Zhang, Lutao Gao, Chunhui Bai, Linnan Yang

**Affiliations:** ^1^ College of Big Data, Yunnan Agricultural University, Kunming, China; ^2^ Yunnan Engineering Technology Research Center of Agricultural Big Data, Kunming, China; ^3^ Yunnan Engineering Research Center for Big Data Intelligent Information Processing of Green Agricultural Products, Kunming, China

**Keywords:** cherry, ripeness identification, real-time semantic segmentation, lightweight segmentation model, smart agricultural

## Abstract

**Introduction:**

Accurate identification of cherry maturity and precise detection of harvestable cherry contours are essential for the development of cherry-picking robots. However, occlusion, lighting variation, and blurriness in natural orchard environments present significant challenges for real-time semantic segmentation.

**Methods:**

To address these issues, we propose a machine vision approach based on the PIDNet real-time semantic segmentation framework. Redundant loss functions and residual blocks were removed to improve efficiency, and SwiftFormer-XS was adopted as a lightweight backbone to reduce complexity and accelerate inference. A Swift Rep-parameterized Hybrid (SwiftRep-Hybrid) module was designed to integrate local convolutional features with global Transformer-based context, while a Light Fusion Enhance (LFE) module with bidirectional enhancement and bilinear interpolation was introduced to strengthen feature representation. Additionally, a post-processing module was employed to refine class determination and visualize maturity classification results.

**Results:**

The proposed model achieved a mean Intersection over Union (MIoU) of 72.2% and a pixel accuracy (PA) of 99.82%, surpassing state-of-the-art real-time segmentation models such as PIDNet, DDRNet, and Fast-SCNN. Furthermore, when deployed on an embedded Jetson TX2 platform, the model maintained competitive inference speed and accuracy, confirming its feasibility for real-world robotic harvesting applications.

**Discussion:**

This study presents a lightweight, accurate, and efficient solution for cherry maturity recognition and contour detection in robotic harvesting. The proposed approach enhances robustness under challenging agricultural conditions and shows strong potential for deployment in intelligent harvesting systems, contributing to the advancement of precision agriculture technologies.

## 1 Introduction

Cherry is one of China’s major economic fruit crops (Zhang et al., 2024), with a continuously increasing market demand. However, the harvesting process faces several challenges, including uneven fruit maturity, high labor intensity, and low picking efficiency. Traditional manual harvesting not only requires extensive labor but also often leads to fruit damage, which reduces market value and diminishes consumer satisfaction. Therefore, the development of efficient and intelligent cherry-picking systems has become a critical research focus in the field of agricultural automation (Liu et al., 2024).

In recent years, commonly used sensors in fruit-picking robots include high-resolution cameras, stereo depth cameras, structured-light depth cameras, and Light Detection and Ranging(LiDAR) (Horaud et al., 2016). High-resolution cameras capture rich color, shape, and texture information, facilitating fruit recognition and maturity estimation. They also offer advantages including low cost and flexible installation. However, their image quality is susceptible to degradation under challenging lighting conditions, such as strong light and shadows (Xu et al., 2024). Stereo depth cameras acquire 3D information through disparity calculation but often suffer from low accuracy in textureless or repetitive texture regions (Luhmann et al., 2016). Structured-light cameras measure depth by projecting structured patterns but are highly sensitive to lighting variations, resulting in poor robustness (Zhong et al., 2021; Maru et al., 2020). LiDAR offers strong resistance to light interference and produces high-precision point clouds. However, its low resolution makes it difficult to capture fruit details, and the equipment is relatively expensive (Liu and Zhang, 2021; Saha et al., 2024). In comparison, high-resolution cameras offer greater advantages in image quality, cost-effectiveness, and deployment flexibility. With advances in imaging technologies and deep learning algorithms, recognition accuracy and robustness of high-resolution cameras have significantly improved, particularly in fruit maturity estimation—a function that LiDAR and depth cameras still struggle to perform effectively. In unstructured agricultural environments, visual information acquisition is a key factor affecting the performance of fruit-picking robots (Wang et al., 2022b; Tang et al., 2020). It not only affects target recognition and localization accuracy but also determines the overall system efficiency. Therefore, a camera-based visual perception system, combined with efficient image segmentation and recognition algorithms, provides a reliable technological foundation for intelligent fruit harvesting (Gongal et al., 2015). In addition, machine vision has been widely applied to the autonomous navigation of agricultural robots (Wang et al., 2022a), further emphasizing the crucial role of visual perception in intelligent harvesting systems.

With the rapid advancement of computer vision and deep learning technologies, fruit maturity detection is no longer confined to traditional approaches. Image-based methods have garnered increasing attention in recent years (Ni et al., 2020). Fruit maturity is a critical indicator influencing both harvesting timing and fruit quality. Accurate identification of maturity can effectively guide robotic harvesting systems toward precision picking.

In the field of robotic fruit harvesting, object detection techniques have been widely adopted for fruit identification and localization. (Gai et al., 2023a). proposed an improved YOLO-V4-based deep learning algorithm for cherry maturity detection. (Gai et al., 2023b). developed a self-supervised cherry maturity detection algorithm based on multi-feature contrastive learning to improve the generalization ability of small-object detection networks in complex environments. (Appe et al., 2023). enhanced YOLO-V5 for tomato maturity detection by incorporating the CBAM module. (Halstead et al., 2018). proposed a robotic vision system based on the Faster R-CNN (Poudel et al., 2019) framework for fruit detection, maturity estimation, and tracking. Jing et al (Jing et al., 2024). proposed an improved object detection algorithm, MRD-YOLO, for melon maturity detection. (Zhu et al., 2024). improved YOLO-V7 by integrating three Criss-Cross Attention (CCA) (Huang et al., 2019) modules and a GSConv (Li et al., 2024) module to address the low efficiency in camellia fruit maturity detection.

However, these object detection methods often exhibit limited accuracy in practical applications. Specifically, object detection models generally provide only bounding box information, making it difficult to accurately distinguish maturity regions—particularly under conditions of fruit clustering or partial occlusion. This lack of precision limits the robot’s ability to accurately assess fruit maturity, thereby reducing the effectiveness of harvesting decisions. Moreover, object detection methods are prone to missed detections and false positives when dealing with multi-scale targets and complex backgrounds, further compromising overall system performance. More refined image segmentation methods are urgently needed to enhance the accuracy of maturity recognition.

Deep learning has also demonstrated strong potential in fruit maturity segmentation tasks. (Xie et al., 2024). proposed the ECD-DeepLabv3+ semantic segmentation model, based on DeepLabV3+ (Chen et al., 2018), for detecting and segmenting sugar apples at different maturity levels. (Kim et al., 2022). developed a dual-path semantic segmentation model capable of simultaneously learning strawberry maturity and peduncle localization. (Kang et al., 2024). proposed a machine vision method based on MobileNetV2 (Sandler et al., 2018) and DeepLabV3+, enabling broccoli head detection, pixel-level identification, maturity classification, and precise localization of harvestable heads.

Currently, fruit maturity detection technologies have achieved notable progress in structured cultivation environments. In such environments, fruit trees exhibit regular morphology, fruits are more uniformly distributed, and background interference is minimal, resulting in relatively low segmentation difficulty. However, existing maturity detection methods still face numerous challenges in complex agricultural environments, including lighting variations, cluttered backgrounds, and fruit occlusion, which reduce segmentation accuracy and degrade the performance of automated harvesting systems. In real-world cherry orchards, tree morphology is diverse, fruit distribution is uneven, and occlusion from leaves and other objects is common, significantly increasing the difficulty of maturity segmentation. Therefore, maturity segmentation under unstructured cultivation environments is equally critical.

Based on the above research and literature review, it is evident that limited studies have addressed cherry maturity segmentation in unstructured cultivation environments. Therefore, this study focuses on addressing this gap. To ensure both accuracy and real-time responsiveness of the cherry maturity segmentation system in harvesting robots, the performance of the semantic segmentation model in terms of accuracy and speed is critical. Inspired by the three-branch architecture of the real-time PIDNet framework (Xu et al., 2023), this study proposes a real-time cherry maturity segmentation algorithm based on an enhanced PIDNet architecture. The algorithm takes PIDNet as the baseline and incorporates the lightweight SwiftFormer-XS (Shaker et al., 2023) as the backbone network. The structure is further optimized by removing redundant loss functions and residual blocks, thereby significantly reducing model complexity and improving inference speed. A SwiftRep-Hybrid module is introduced to re-parameterize each SwiftFormer block in the original SwiftFormer-XS, thereby improving segmentation accuracy without compromising FPS performance. Additionally, a Light Fusion Enhance (LFE) module is incorporated to enhance boundary information processing. The proposed approach improves segmentation accuracy while preserving a lightweight model architecture. The main contributions of this study are summarized as follows:

1. The original PIDNet architecture is optimized by replacing the backbone network and eliminating redundant loss function computations, which significantly reduces model complexity and enhances inference speed.
2. A Swift Rep-parameterized Hybrid (SwiftRep-Hybrid) module is proposed, combining local feature extraction through convolution with global context modeling via Transformer mechanisms. This module significantly enhances the model’s ability to capture fine details in complex image scenes and improves segmentation performance in unstructured cultivation environments.
3. A Light Fusion Enhance (LFE) module is introduced, incorporating a bidirectional enhancement mechanism and bilinear interpolation to effectively mitigate the effects of occlusion, blurriness, and illumination variation in complex environments.
4. A dedicated post-processing module for semantic segmentation is developed, enabling the visualization of cherry maturity and spatial information through a PyQt5-based interface, thereby demonstrating the practical application of semantic segmentation in maturity recognition.

## 2 Materials and methodology

### 2.1 Overall process

**Figure 1:**
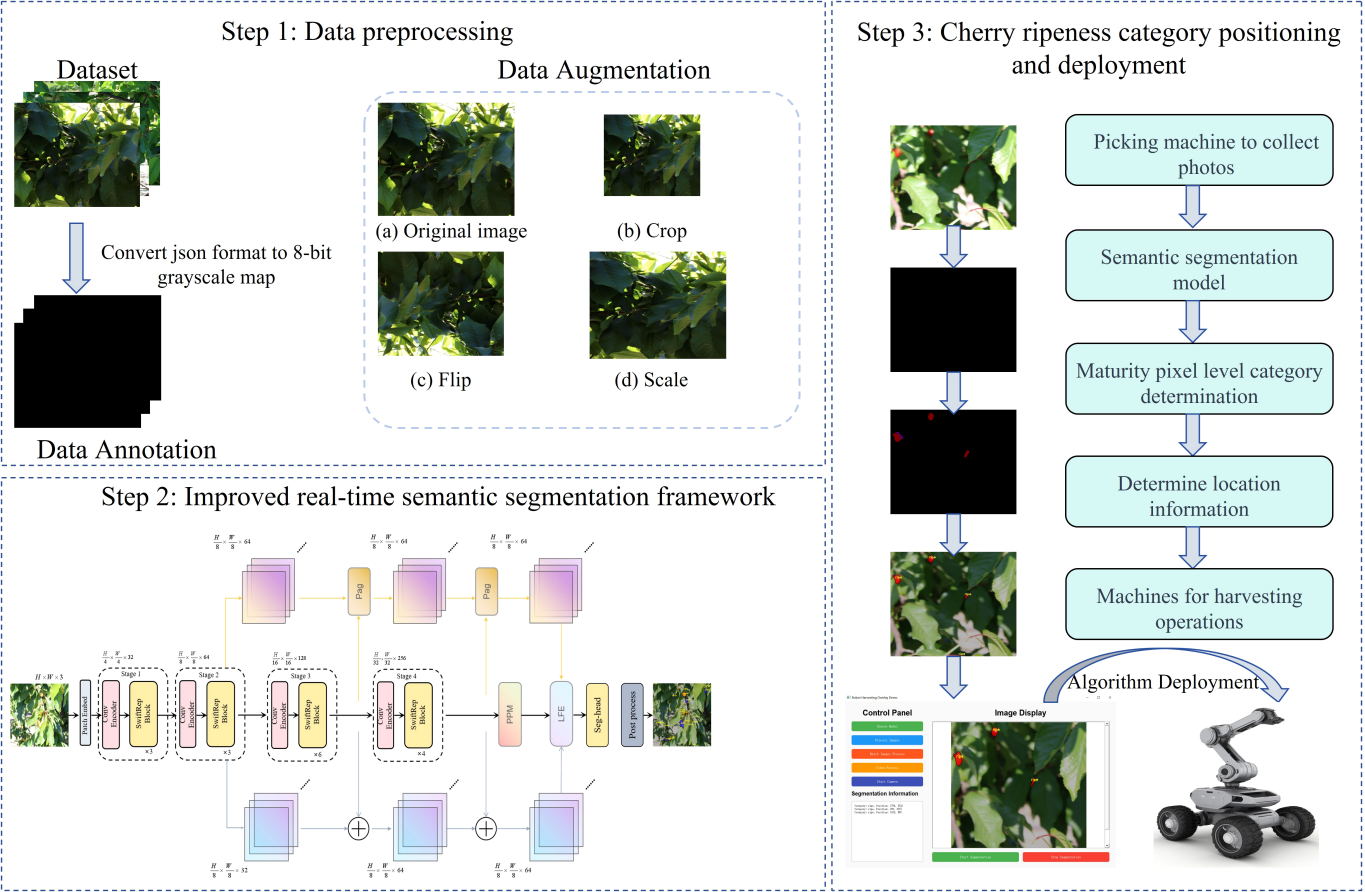
Overall workflow.

The overall workflow of this study is illustrated in **Figure 1** and comprises three main stages: (1) data preprocessing; (2) development of an improved semantic segmentation model for pixel-level recognition of cherry maturity; (3) design of a post-processing module for visualizing ripening categories and localization of cherries.

### 2.2 Dataset

In recent years, several cherry datasets (e.g.CherryBBCH81 (Apeinans et al., 2024), CherryBBCH72 (Kodors et al., 2024), and the SweetCherry dataset (Li et al., 2022) have been primarily utilized for object detection tasks. These datasets typically provide bounding box annotations of cherries rather than pixel-level segmentation labels, limiting the model’s ability to accurately delineate fruit boundaries in complex scenarios. Particularly in agricultural environments characterized by heavy occlusion, significant lighting variation, and diverse cherry morphology, object detection methods are prone to misclassifying background and target regions, thereby hindering subsequent fine-grained analysis and real-world deployment.

**Figure 2:**
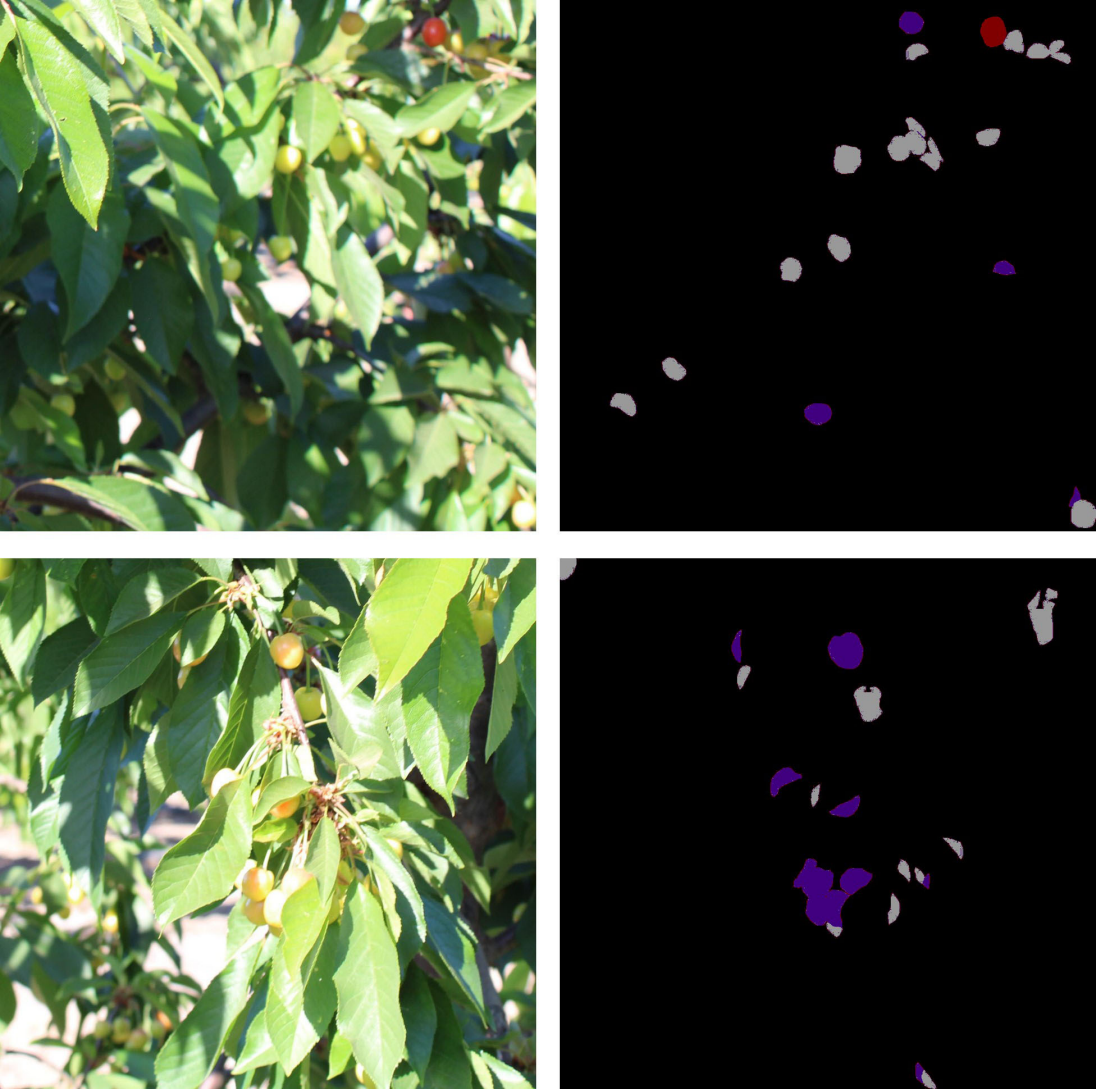
Few examples of cherry CO Dataset-ripeness dataset.

To address this issue, this study employs the Cherry CO Dataset-ripeness (Cossio-Montefinale et al., 2024), comprising 3,006 high-resolution images (1328×1328) with annotations for over 15,000 individual cherries. The dataset categorizes cherries into three maturity stages: green (early development), unripe (yellow to orange), and ripe (various shades of red). The dataset was collected under diverse conditions, including sunny and cloudy weather, various time periods, and multiple viewpoints, accounting for challenges such as lighting variations, occlusion, size differences, and focal shifts. These factors reflect the inherent complexity of agricultural environments and offer a diverse set of training and testing samples for this study. Detailed dataset specifications are provided in **Tables 1**, **2**, while **Figure 2** presents representative examples from the Cherry CO Dataset-ripeness. In **Figure 2** Ripe fruits are represented by dark red, unripe fruits by dark purple-blue, and green fruits by medium gray.

**Table 1:** Distribution of the number of pictures.

Dataset split	Number of images
Train	1804
Validation	601
Test	601

**Table 2:** The quantities of each category.

Category	Number of annotations
Rip	6684
Unripe	4211
Green	3524

### 2.3 Maturity segment network structure

**Figure 3:**
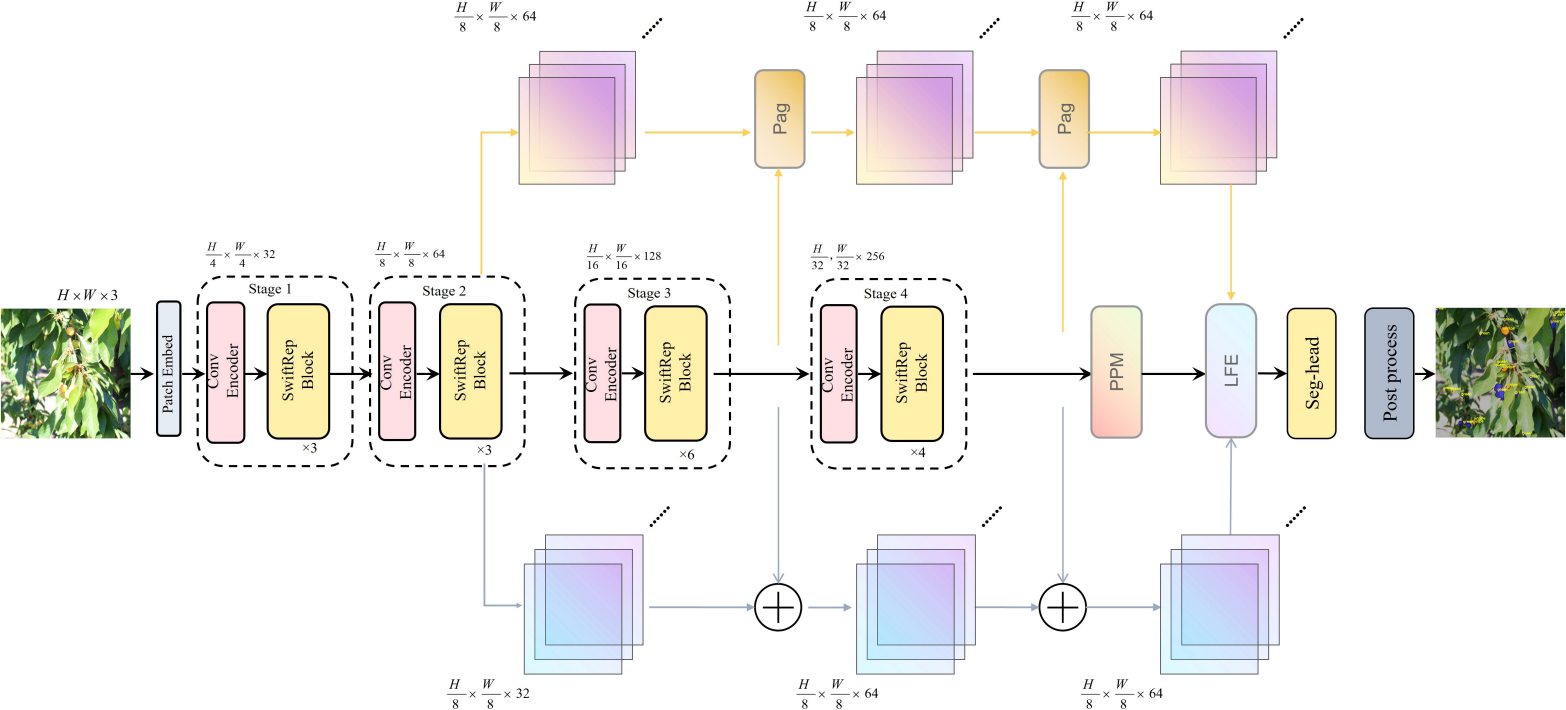
The network structure diagram we proposed.

PIDNet serves as the foundational architecture, upon which multiple structural enhancements are introduced. PIDNet is a real-time semantic segmentation network with a three-branch architecture that processes multi-scale features via parallel intermediate decoders, thereby improving model stability and generalization capability. Specifically, PIDNet introduces multiple intermediate decoders after the backbone, each responsible for feature fusion and segmentation tasks at different hierarchical levels. This architectural design not only enhances the models ability to capture multi-scale information but also improves segmentation performance in complex environments. In addition, PIDNet leverages an effective feature fusion mechanism to better capture image details and contextual relationships, thereby further enhancing segmentation accuracy. As illustrated in **Figure 3**.

To clarify the architectural design and the contribution of each component in our model, **Table 3** summarizes the key differences between our method and representative real-time segmentation architectures, including PIDNet, SwiftFormer-XS, and BiSeNetV2. Specifically, we adopt the lightweight Transformer-based SwiftFormer-XS as the backbone and adapt the RepViTBlock for efficient multi-stage feature extraction. Two novel modules are proposed: the SwiftRep-Hybrid module for local-global feature fusion, and the LFE (Light Fusion Enhance) module for robust feature enhancement under occlusion and illumination variation. Additionally, a lightweight post-processing module is introduced to visualize the maturity classification results more clearly, facilitating potential deployment in robotic harvesting platforms.

**Table 3:** Comparison of model components and innovations.

Component	PIDNet	BiSeNetV2	Ours	Category
Backbone	CNN (three-branch)	Dual-branch CNN	SwiftFormer-XS	Adopted
Lightweight Module	–	Detail branch	RepViTBlock	Adapted
Local-Global Fusion	Cross-path fusion	Feature fusion	SwiftRep-Hybrid	Proposed
Feature Enhancement	Attention-based	Aggregation node	LFE module	Proposed
Post-processing	Simple upsample	–	Maturity-level refinement	Adapted
Target Application	Urban scenes	Real-time parsing	Cherry segmentation	Task-adapted

#### 2.3.1 Change the backbone network

When performing image segmentation tasks on the cherry dataset, the original PIDNet employs ResNet18 (He et al., 2016) as its backbone network. ResNet18 primarily relies on stacked convolutional layers for feature extraction, which are effective at capturing local features. However, global contextual information is critical in semantic segmentation tasks. In particular, under conditions such as occlusion, lighting variation, and densely distributed fruits, the lack of global context often leads to unstable segmentation results. Although ResNet18 is lighter than ResNet50 (He et al., 2016), its standard convolutional operations still incur substantial computational cost, which may hinder real-time inference on robotic platforms. Moreover, ResNet18 depends on deeper layers to extract high-level semantic features, resulting in longer inference latency and reduced decision-making efficiency. In robotic cherry harvesting scenarios, mature cherries vary significantly in size and are often partially occluded by leaves. ResNet18 performs classification on low-resolution deep feature maps, where small objects may be lost during downsampling, resulting in suboptimal segmentation performance for small targets.

**Figure 4:**
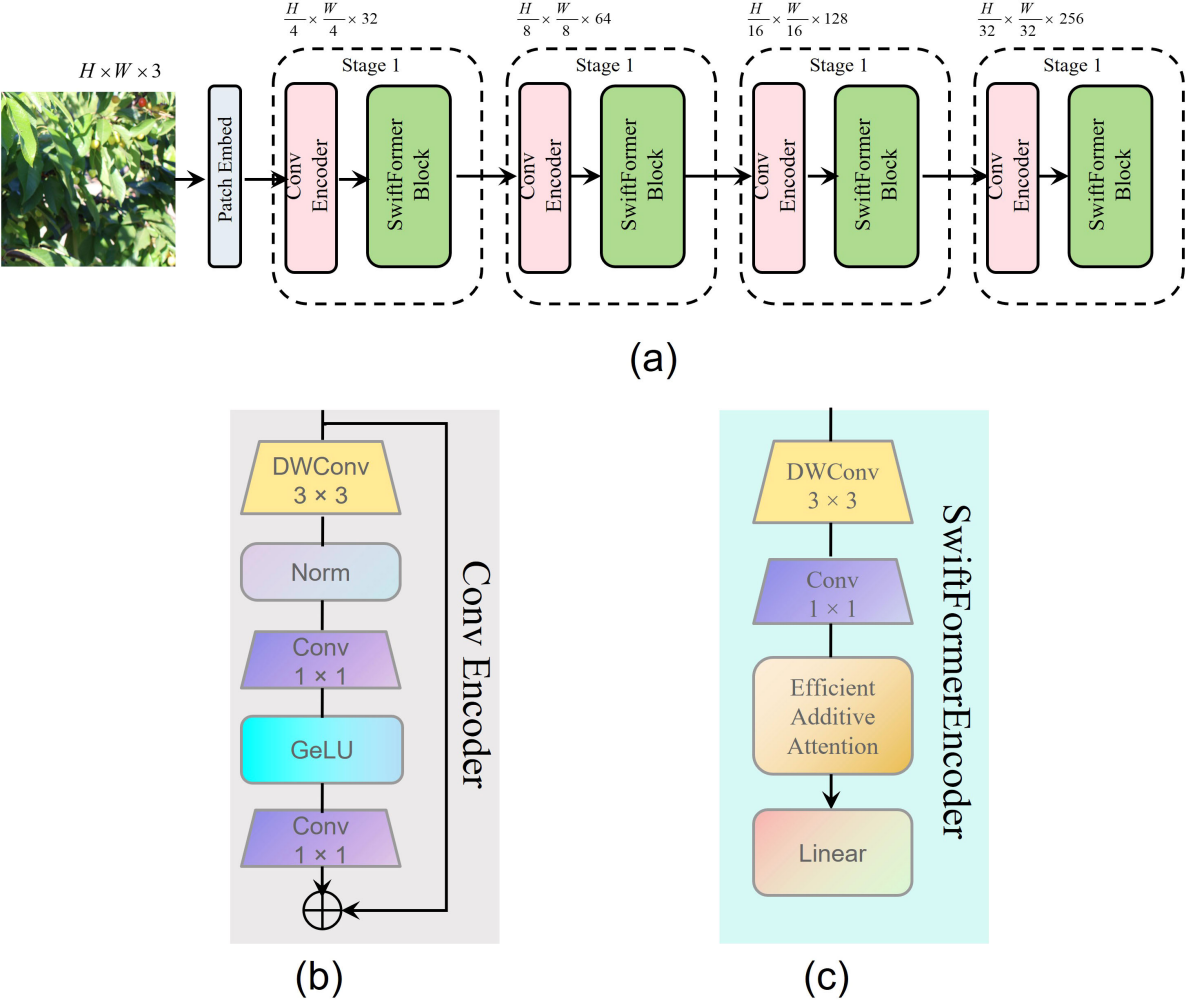
**(a)** SwiftFormer structure. **(b)** Conv Encoder. **(c)** SwiftFormerEncoder.

The cherry dataset includes images across various growth stages, lighting conditions, and complex backgrounds, imposing high demands on segmentation accuracy and computational efficiency. To address these challenges, SwiftFormer-XS is adopted to replace ResNet18, aiming to enhance feature extraction capacity while reducing computational complexity. SwiftFormer-XS integrates local feature extraction via convolution and global contextual modeling via Transformer mechanisms (Vaswani et al., 2017), enabling more comprehensive multi-scale representation under varying lighting and occlusion conditions. Compared to ResNet18, SwiftFormer-XS is composed of four stages, each consisting of a Conv Encoder (**Figure 4b**) and a SwiftFormer Block (**Figure 4c**), as illustrated in **Figure 4a**. For the input image, SwiftFormer-XS adopts a lightweight patch embedding module to enable efficient feature extraction while minimizing redundant computations. This is achieved by integrating depthwise separable convolutions (SIfre and Mallat, 2014) within both the Conv Encoder and the SwiftFormer Block. Furthermore, the SwiftFormer Block employs an Efficient Additive Attention mechanism to effectively capture long-range dependencies, thereby enhancing the overall representation capability of the network. Given the complex backgrounds and varying fruit sizes in agricultural scenarios, the hybrid feature extraction capability of SwiftFormer-XS enhances maturity recognition accuracy while reducing computational overhead, making it well-suited for deployment on resource-constrained robotic platforms.

Additionally, the auxiliary loss functions in the original PIDNet architecture are removed in this study to reduce redundant computations and optimize inference speed. In the original PIDNet, multiple auxiliary loss terms are used during training to enhance the feature representation in intermediate layers. However, experimental results on our dataset indicate that these auxiliary losses offer limited benefits for final maturity segmentation performance, while incurring substantial computational cost. As shown in **Table 4**, the overall computation is reduced from 39.17 GFLOPs to 38.99 GFLOPs, reflecting a modest but meaningful reduction that contributes to faster inference. As a result, only the final main loss function is retained to directly optimize the segmentation output, thereby reducing computational load and improving inference time. This modification enhances the model’s real-time performance and makes it more suitable for deployment on mobile computing units or embedded platforms.

**Table 4:** Ablation experiments conducted on the CherryCO-Ripeness val set to evaluate the impact of different components.

Backbone	RepBlock	LFE	MIoU (%)	MPA (%)	Params (M)	Model Size (MB)	FPS (f/s)	FLOPs (G)
×	×	×	70.19	80.04	7.6	29.08	100.02	39.17
✓	×	×	71.64	81.07	**5.4**	**20.86**	**109.89**	**38.99**
✓	✓	×	72.00	82.29	5.6	21.73	105.32	40.62
✓	✓	✓	**72.20**	**82.48**	5.9	22.86	104.63	41.64

Bold represents the best result. MIoU and MPA are in percentage (%), Params in millions (M), Model Size indicates the file size (MB), and FPS in frames per second (f/s), FLOPs refer to floating point operations (GigaFLOPs).

Therefore, this study adopts SwiftFormer-XS as the backbone network and removes unnecessary auxiliary loss computations, effectively reducing model parameters, optimizing computational complexity, and improving inference speed. The enhancement significantly boosts segmentation accuracy while improving robustness to lighting variation, occlusion, and scale differences.

#### 2.3.2 Improve SwiftFormer network

In image segmentation tasks, particularly when processing cherry image data, the SwiftFormer-XS network encounters challenges in maintaining both segmentation accuracy and computational efficiency in complex scenarios. Although SwiftFormer-XS enhances the model’s representational capacity through progressive feature extraction at multiple stages, its original architecture struggles to capture long-range dependencies effectively and demonstrates relatively low inference efficiency, particularly when processing large-scale cherry image datasets. To address these limitations, this study proposes an optimization strategy based on RepViTBlock, which integrates re-parameterization techniques with Vision Transformer principles by embedding RepViTBlock (Wang et al., 2024) into each stage of SwiftFormer-XS.

**Figure 5:**
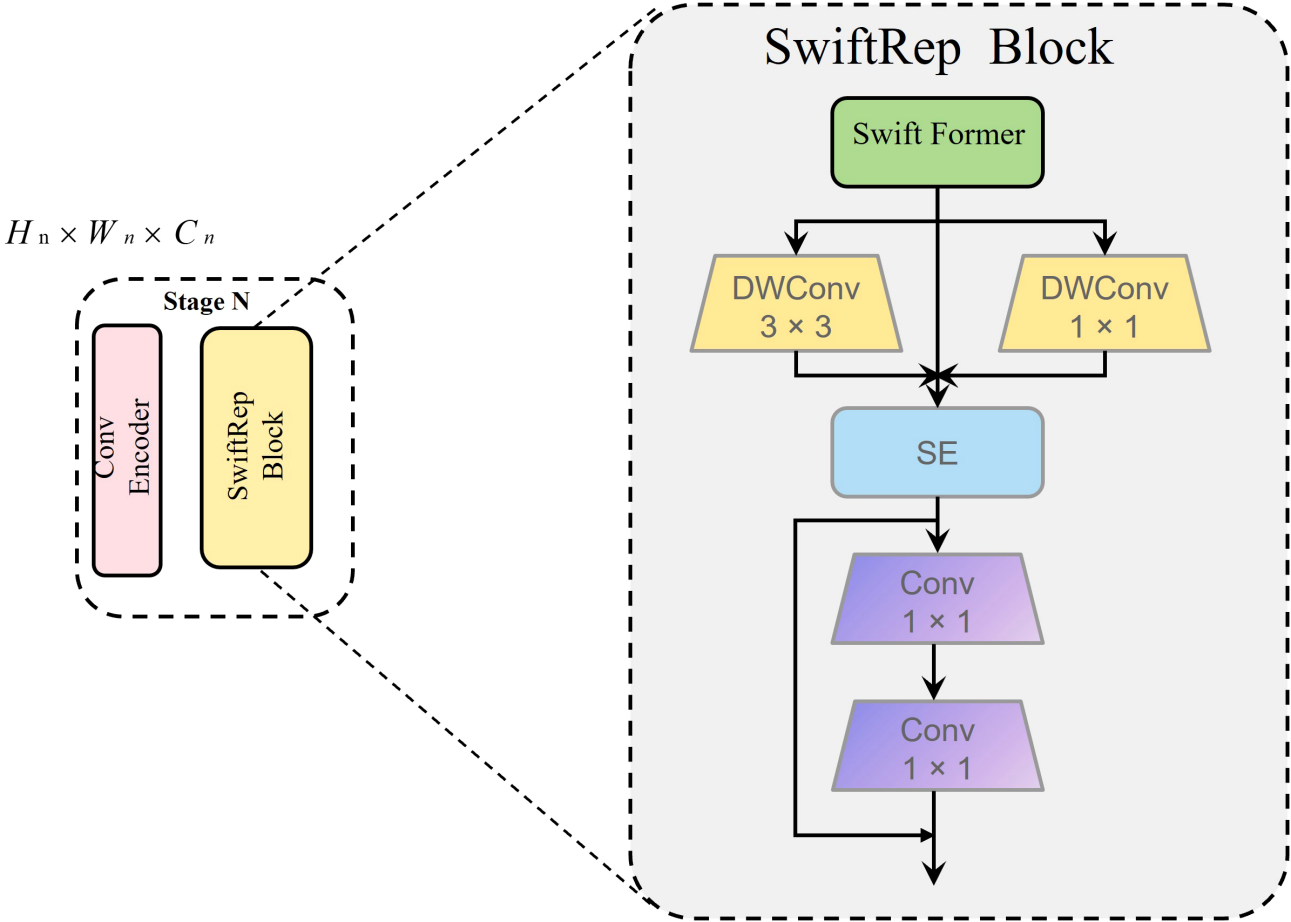
The SwiftRep Block network structure.


As illustrated in **Figure 5**, the SwiftRep Block first processes the input feature *X* through a SwiftFormer module to capture global dependencies, producing the global feature *F_g_* (Equation 1):

(1) $$F_{g}=\text{SwiftFormerBlock}\left(X\right)$$

To extract local detailed information, two parallel branches apply depthwise separable convolutions with kernel sizes of 3 × 3 and 1 × 1 on *F_g_*, generating local features $F_{3\times 3}$ (Equation 2) and $F_{1\times 1}$ (Equation 2), respectively:


(2) $$F_{l}^{3\times 3}={\text{DWConv}}_{3\times 3}\left(F_{g}\right),\;F_{l}^{1\times 1}={\text{DWConv}}_{1\times 1}\left(F_{g}\right)$$

These two local feature branches are then fused by element-wise summation to obtain the combined local feature *F_l_* (Equation 3):


(3) $$F_{l}=F_{l}^{3\times 3}+F_{l}^{1\times 1}$$

During inference, structural re-parameterization merges the dual-branch convolution into a single convolution kernel, significantly accelerating the inference speed.

Next, a Squeeze-and-Excitation (SE) module adaptively recalibrates the channel-wise feature responses of *F_l_* (Equation 4):


(4) $$F_{se}=\text{SE}\left(F_{l}\right)$$

Subsequently, two consecutive 1×1 convolution layers perform efficient feature fusion and dimensionality adjustment, resulting in refined feature representations *F*_1_ (Equation 5) and *F*_2_ (Equation 5):


(5) $$F_{1}={\text{Conv}}_{1\times 1}^{\left(1\right)}\left(F_{se}\right),\;F_{2}={\text{Conv}}_{1\times 1}^{\left(2\right)}\left(F_{1}\right)$$

Finally, the refined local feature *F*_2_ is fused with the feature *F_se_* via element-wise addition to produce the output feature *F*_out_ (Equation 6):


(6) $$F_{\text{out}}=F_{2}+F_{se}$$

This design effectively alleviates the computational bottleneck of SwiftFormer-XS when processing large-scale datasets while the embedded Transformer structure excels at capturing long-range dependencies. Consequently, the model better perceives global information in cherry images, which is beneficial for accurately identifying the position and maturity status of individual cherries in clusters with varying densities, thereby mitigating adhesion issues during segmentation and meeting real-time requirements.

By integrating RepViTBlock after each stage of SwiftFormer-XS, the model’s multi-scale feature extraction capability is progressively refined, facilitating learning from global distribution to local details. This enables the model to gain a deeper understanding of the semantic structure in cherry images. This architectural innovation significantly enhances the performance of the PIDNet network in cherry image segmentation tasks, particularly improving segmentation accuracy, completeness, and boundary smoothness in complex scenarios. It also reduces the occurrence of mis-segmentation. The detailed network architecture is illustrated in **Figure 5**.

#### 2.3.3 Light-Fusion Enhance module

In the existing Light-Bag (Xu et al., 2023) module, although feature fusion is effectively handled through convolution operations and batch normalization(BatchNorm), several limitations persist. First, Light-Bag adopts a simple weighted feature fusion strategy based on edge attention maps, which enables limited channel-wise feature integration. However, it lacks sufficient capacity to capture fine-grained image details, particularly under complex environmental conditions such as occlusion and lighting variations, thereby limiting the model’s overall performance. Second, Light-Bag does not fully exploit multi-scale features, making it prone to overlooking critical semantic and detailed information in the image, which negatively impacts segmentation accuracy. Finally, although the Light-Bag module is lightweight, its structural simplicity may limit its ability to provide sufficient feature enhancement and robustness in complex segmentation tasks. To address these issues, this study proposes the Light Fusion Enhance(LFE) module.

**Figure 6:**
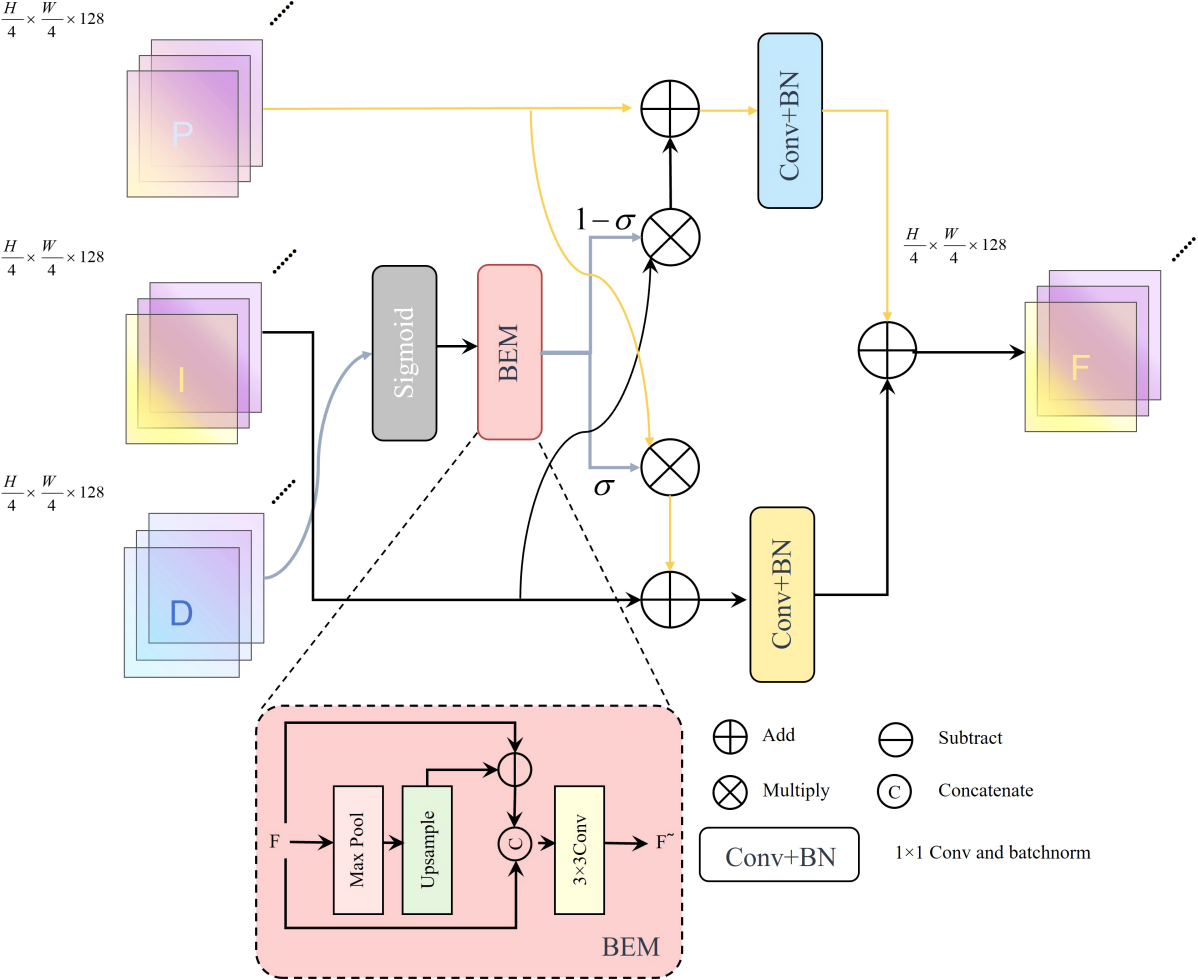
The LFE module structure.

First, the feature map D is activated by a Sigmoid function to generate an initial edge-guided map, which is then fed into the Boundary Enhancement Module(BEM) (Ali et al., 2025) to produce an edge-aware attention map, denoted as *edge_att_*, as illustrated in Equation 7. The BEM enhances structural detail by computing local contrast. Specifically, it processes *D* via a contrastive branch, where *D*′ is the intermediate edge feature map obtained by a shallow convolution. Then, MaxPool(*D*′) captures coarse edge patterns, which are upsampled and subtracted from *D*′ to highlight local differences. This result is concatenated with *D*′ and passed through a 3 × 3 convolution to generate the enhanced edge feature *F_BEM_* (Equation 8). The edge attention *edge_att_* is then used to guide information exchange between two branches: the position branch *P* and the intensity branch *I*, both derived from *D*. These represent spatial and texture features, respectively. The updated features *P*′ and *I*′ are computed by blending one branch into the other, weighted by *edge_att_* (Equations 9, 10). This mechanism allows edge-sensitive areas to integrate more positional detail (via *P*) and context (via *I*). Subsequently, both *P*′ and *I*′ are projected via 1 × 1 convolutions to obtain *P*′′ and *I*″ (Equation 11) for dimension reduction and alignment. The final fused feature *F* is computed by element-wise summation of the two projected features (Equation 12), forming the output of the Light Fusion Enhancement (LFE) module,as shown in the **Figure 6**.

LFE integrates the strengths of both the Light-Bag and BEM modules, incorporating a bidirectional enhancement mechanism within a lightweight architecture. Moreover, LFE improves the model’s perception of cherry details and morphological features by fusing and enhancing multi-scale information, thereby significantly improving segmentation accuracy and robustness in maturity segmentation tasks. Compared to Light-Bag, LFE not only maintains high computational efficiency but also delivers superior performance in real-world applications.


(7) $$edge_{att}=\text{BEM}\left(\text{Sigmoid}\left(D\right)\right)$$

(8) $$F_{BEM}={\text{Conv}}_{3\times 3}\left(\text{Concat}\left(D^{\prime },\text{Upsample}\left(\text{MaxPool}\left(D^{\prime }\right)\right)-D^{\prime }\right)\right)$$

(9) $$P^{\prime }=\left(1-edge_{att}\right)\cdot I+P$$

(10) $$I^{\prime }=edge_{att}\cdot P+I$$

(11) $$P^{\prime \prime }={\text{Conv}}_{1\times 1}\left(P^{\prime }\right),\;I^{\prime \prime }={\text{Conv}}_{1\times 1}\left(I^{\prime }\right)$$

(12) $$F=P^{\prime \prime }+I^{\prime \prime }$$

### 2.4 Loss function

In fruit maturity classification and segmentation tasks, class imbalance frequently arises due to uneven distribution of samples across different maturity levels, leading to a decline in overall model performance. For instance, in the application of cherry-picking robots, datasets often contain a dominant proportion of ripe cherries, while green and unripe samples are underrepresented. If the model predominantly focuses on the majority class during training, it may misclassify rare categories such as unripe fruits, leading to incorrect picking decisions and potential economic losses. Moreover, cherries are small objects, and occlusion by leaves frequently occurs during the harvesting process. Therefore, this study adopts a combined loss strategy by integrating Online Hard Example Mining (OHEM) (Shrivastava et al., 2016) with standard cross-entropy loss (Krizhevsky et al., 2012) to encourage the model to focus more on underrepresented samples and improve overall segmentation performance. Additionally, weighted binary cross-entropy (CE) loss and boundary loss (Kervadec et al., 2019) are incorporated to emphasize boundary regions and enhance the feature representation of small objects, thereby improving segmentation precision.


(13) $$l_{CE}=-y\text{log }(p)$$

(14) $$l_{Hard}=l_{CE}l_{CE}>0.7$$

(15) $$L_{ohemCE}=\frac{1}{N}\sum_{n=0}^{N-1}l_{Hard}$$

(16) $$L_{BD}=-\sum_{i,j}\{1:b_{i}>m\}\left(a_{i,j}\text{log }s_{i,j}\right)$$

Where *l_CE_* (Equation 13) denotes the cross-entropy loss, y represents the ground truth label for each pixel, and p denotes the predicted probability for the corresponding pixel. *l_Hard_* (Equation 14) indicates the loss for hard examples, *L_ohemCE_* (Equation 15) refers to the combined loss function that integrates Online Hard Example Mining (OHEM) and Cross-entropy loss(CE). N denotes the number of selected hard examples. *L_BD_* (Equation 16) represents the boundary loss function, where m is a predefined threshold, and *b_i_*, *a_i,j_*, and *s_i,j_* refer to the boundary head output, ground truth segmentation, and predicted segmentation output for the i-th pixel in class j, respectively. Accordingly, the final loss function is defined as follows (Equation 17):


(17) $$L={\text{λ}}_{0}l_{CE}+{\text{λ}}_{1}L_{\text{ohemCE}}+{\text{λ}}_{2}L_{BD}$$

**Figure 7:**
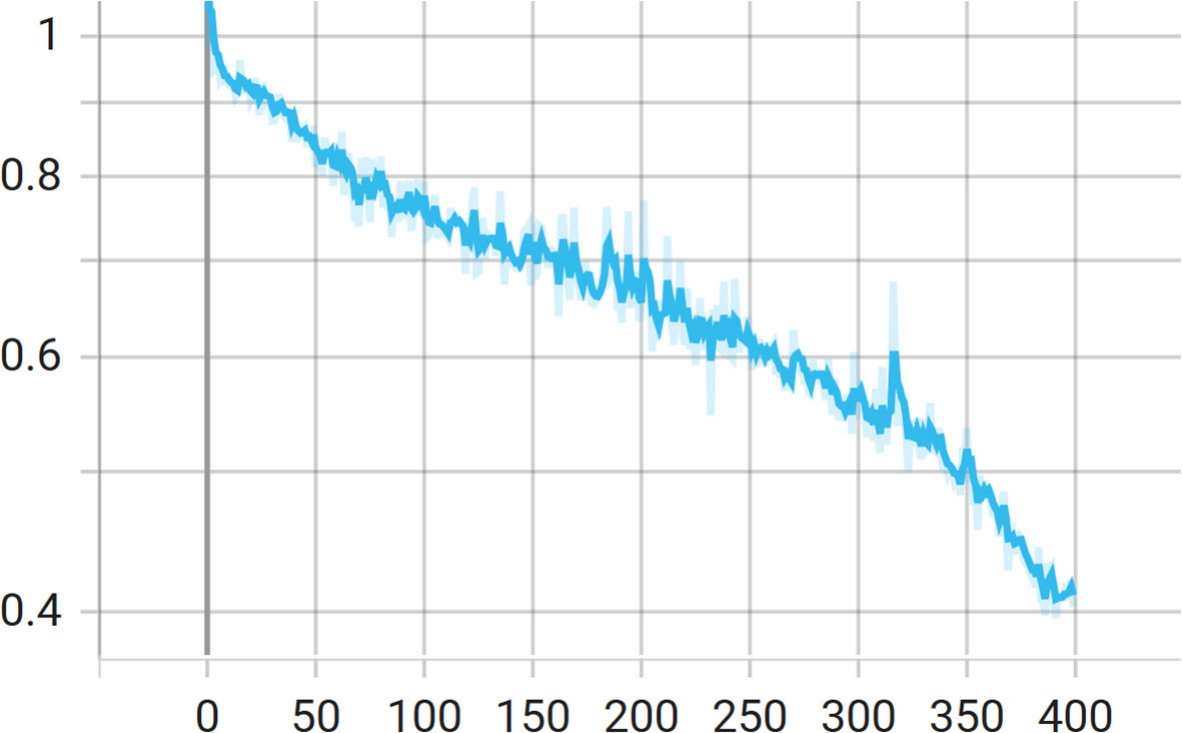
Training loss over epochs.

Based on previous experience, the loss function parameters in our network training are set as *λ*_0_ = 20, *λ*_1_ = 1, *λ*_2_ = 1 and m=0.8,where Ldenotes the total loss of all training samples. *λ*_0_ is assigned a larger value (set to 20) to prioritize accurate pixel-wise segmentation, which is the primary task of our network. This ensures that the cross-entropy loss strongly influences gradient updates. *λ*_1_ (set to 1) governs the influence of the OHEM loss. It ensures that hard-to-classify pixels are emphasized without dominating the training process. *λ*_2_ (also set to 1) controls the contribution of the boundary-aware loss, which helps the model learn sharp edges and object contours, especially important in distinguishing overlapping or partially occluded cherries. The variation of the loss during the training process is illustrated in **Figure 7**.

### 2.5 Evaluation method of prediction performance

We conducted comparative experiments under a controlled hardware configuration and fixed parameter settings, as reported in (Bai et al., 2024). The standard evaluation metrics include Pixel Accuracy (PA) (Equation 18), Category Pixel Accuracy (CPA) (Equation 19), Mean Pixel Accuracy (MPA) (Equation 20), and Mean Intersection over Union (MIoU) (Equation 21). Assuming there are n classes, *p_ii_* denotes the number of pixels correctly classified for class i, and *p_ij_* represents the number of pixels that belong to class i but are misclassified as class j. The definitions of the evaluation metrics are as follows:


(18) $$PA=\frac{\sum_{i=0}^{n}p_{ii}}{\sum_{i=0}^{n}\sum_{j=0}^{n}p_{ij}}$$

(19) $$CPA_{i}=\frac{p_{i}}{\sum_{j=0}^{n}p_{ij}}$$

(20) $$MPA=\frac{1}{n+1}\sum_{i=0}^{n}\frac{p_{ii}}{\sum_{j=0}^{n}p_{ij}}$$

(21) $$MIoU=\frac{1}{n+1}\sum_{i=0}^{n}\frac{p_{ii}}{\sum_{j=0}^{n}p_{ij}+\sum_{j=0}^{n}p_{ji}-p_{ii}}$$

### 2.6 Evaluation method of prediction performance

This study proposes a post-processing method based on transparent overlays of segmentation results, aiming to achieve accurate localization and classification of cherries at different maturity stages. Traditional approaches typically use minimum enclosing circles (Kang et al., 2024) to mark target positions. In contrast, the proposed method applies semi-transparent color masks directly over the predicted segmentation map, visually highlighting the shapes of target regions. Specifically, for each candidate region, a predefined overlay color is selected based on its classification result, and the overlay effect is achieved through weighted blending. The coordinates of cherries are also displayed using a PyQt5-based interface. This method not only supports real-time monitoring but also provides enriched visual information for robotic path planning.

**Figure 8:**
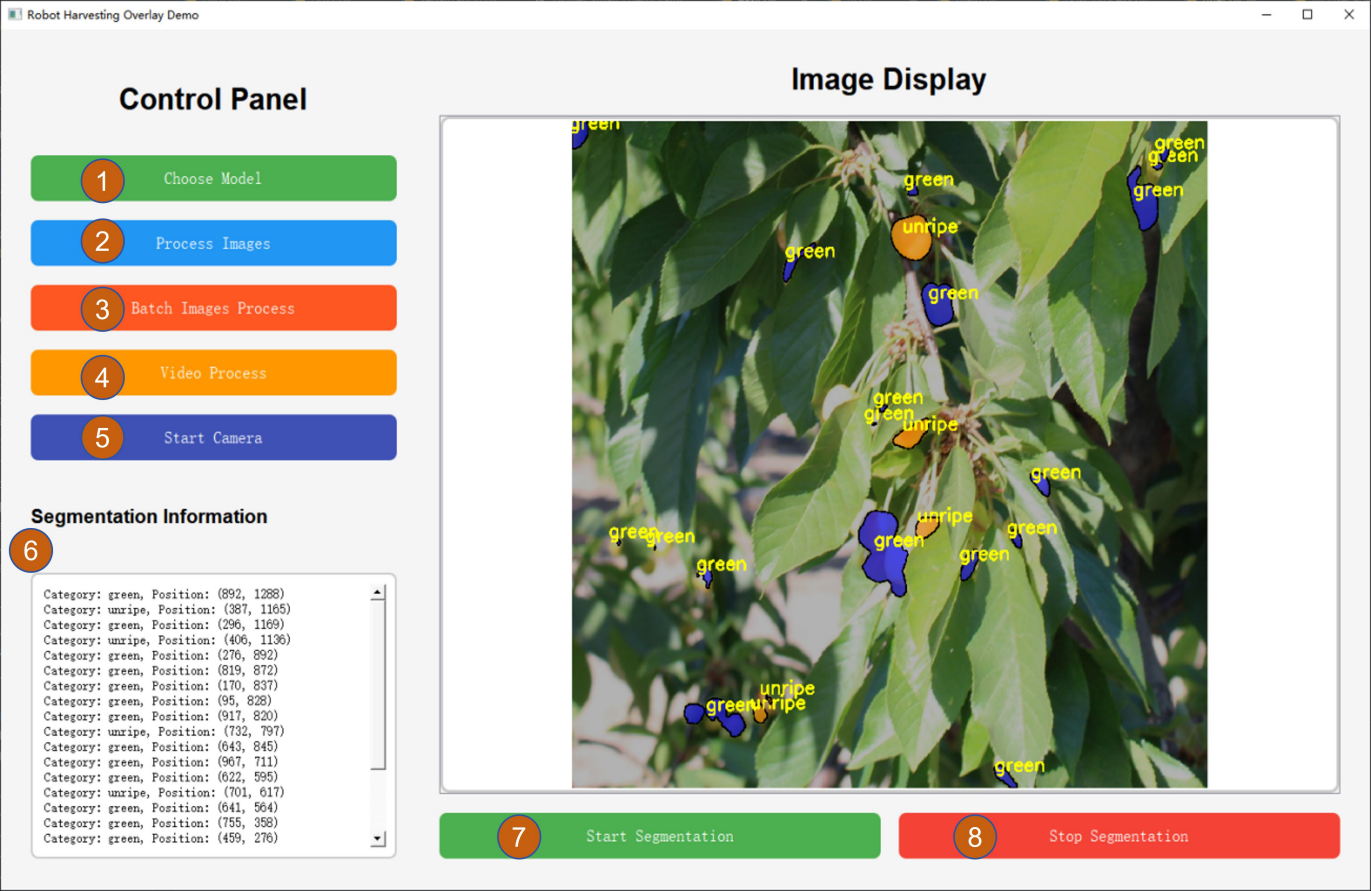
Visual interface for cherry ripeness recognition and classification (1) Selection of trained models (2) Processing a single image (3) Batch processing of images (4) Processing Video (5) Turn on the camera (6) Showing the coordinates of cherries at different levels of ripeness (7) Start Segmentation processing (8) Stop.

To improve system operability and real-time visualization, a graphical user interface (GUI) based on PyQt5 is designed for displaying and interacting with post-processing results, as illustrated in **Figure 8**. The interface allows users to load the desired model and corresponding weights, perform inference, visualize the processed images in real time, and display positioning information in a side panel. It supports functionalities such as model selection, batch image processing, video input, real-time camera feed, and coordinate display. This design improves system interactivity and user-friendliness, making it particularly suitable for farm operators to monitor and adjust the picking process in real time. The GUI is planned to be integrated into the human-machine interaction interface of the cherry-picking robot in future deployments.

### 2.7 Training details

The experiments were conducted on a server running the Linux x86–64 kernel with Ubuntu 20.04.6 LTS as the operating system. The hardware configuration included a 16-core Intel^®^ Xeon^®^ Platinum 8481C processor and an NVIDIA GeForce RTX 4090D GPU with 24 GB of VRAM. The model was implemented using the PyTorch deep learning framework (version 1.12.0), with Python 3.8.0 and CUDA 11.6.

During training, the batch size was set to 6, with six images fed into the model per iteration. The images were randomly cropped to 1312×1312 pixels and randomly scaled within a range of [0.75, 2]. The model was trained for 400 epochs, with 300 iterations per epoch. The Stochastic Gradient Descent(SGD) optimizer was used for parameter updates, with an initial learning rate of 1e-2, a momentum of 0.9, and a weight decay coefficient of 5e-4.A polynomial learning rate decay strategy was adopted, with a power parameter of 0.9. Data augmentation techniques included random horizontal flipping, random cropping, and random scaling to improve model generalization ability. During testing, the batch size was also set to 6, and ablation experiments and comparative experiments on test sets.

## 3 Experiment

### 3.1 Ablation experiment

To verify the effectiveness of the proposed improvements, ablation experiments were conducted to evaluate the contribution of each individual component. PIDNet was used as the baseline model, and improvements were introduced incrementally. The first group used the baseline model(PIDNet). In the second group, the ResNet18 backbone is replaced with a SwiftFormer-based backbone, and redundant loss functions are removed to optimize the overall network architecture. The third group further re-parameterized the backbone network by replacing the original SwiftFormer blocks with Swift Rep-parameterized Hybrid Blocks. The fourth group introduced a Lightweight Fusion Enhancement(LFE) module to replace the original Border Attention Guided Fusion module. MIoU, MPA, and FPS were used as evaluation metrics. In the table, MB represents the storage space required by the model. The ablation results are presented in **Table 4**.

As shown in **Table 4**, each proposed improvement contributes to a notable enhancement in model performance. In the first group, the baseline model achieved an MIoU of 70.19%, an MPA of 80.04%, and an inference speed of 100.02 FPS.In the second group, replacing the ResNet18 backbone with SwiftFormer and eliminating redundant loss functions led to an optimized network structure. It not only improves the MIoU and MPA to 71.64% and 81.07%, but also increases the speed to 109.89 f/s, and at the same time makes drop excessive number of parameters, which verifies that the optimized network structure based on the optimized network has high performance. In the third group, the backbone network was further optimized by re-parameterizing SwiftFormer blocks with Swift Rep-parameterized Hybrid Blocks, which integrate local feature extraction and global context modeling. This design improves the mean Intersection over Union (MIoU) with minimal increase in model parameters. Notably, the MIoU and mean Pixel Accuracy (MPA) reach 72.00% and 82.29%, respectively, while maintaining nearly unchanged inference speed. In the fourth group, the Lightweight Fusion Enhancement(LFE) module was introduced to replace the original pixel attention guidance module. Although a slight decrease in inference speed and a minor increase in model parameters were observed, the real-time requirement was still met. Moreover, the MIoU and macro F1 score increased to 72.20% and 82.48%, respectively.

**Figure 9:**
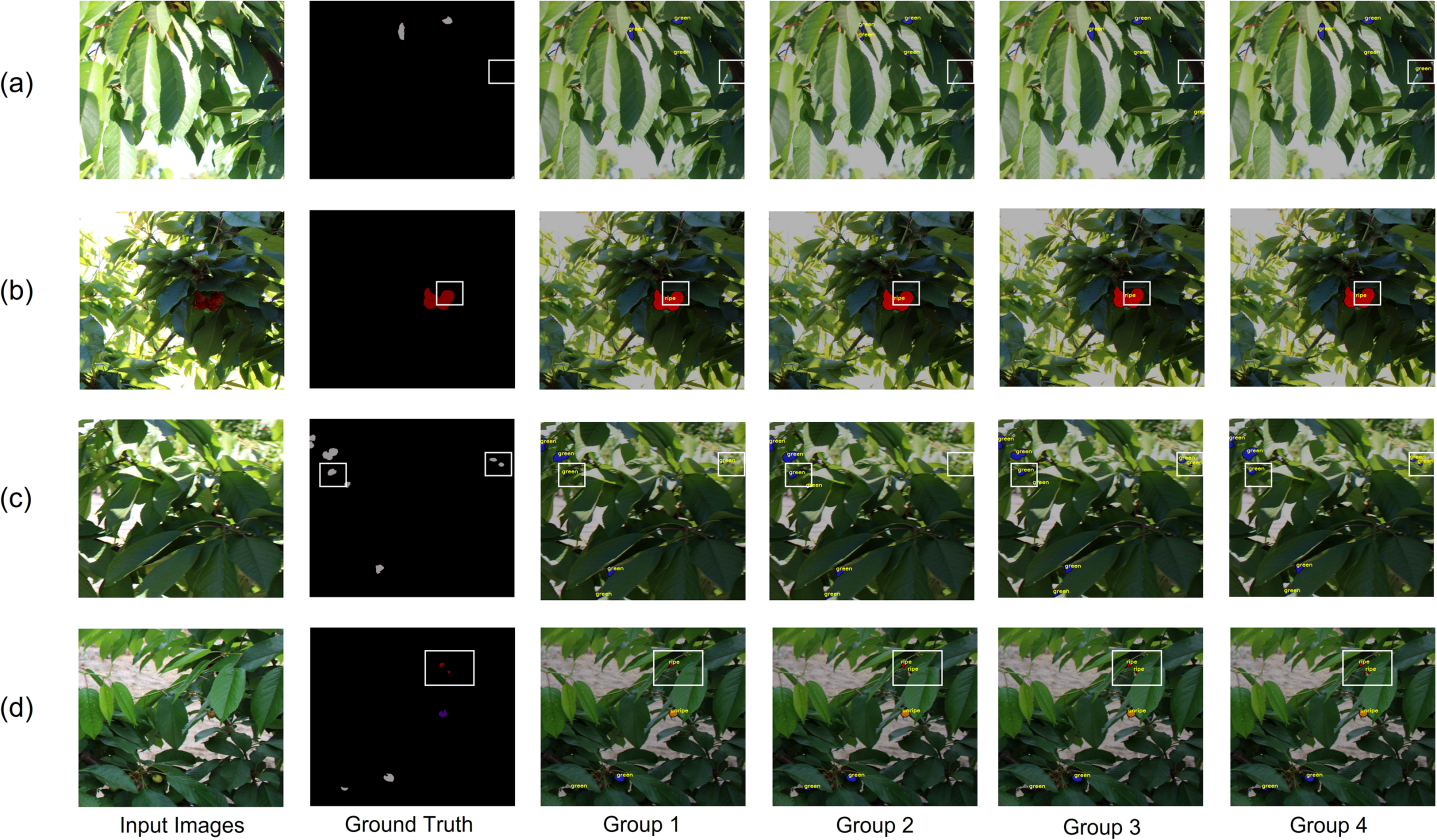
Qualitative results of ablation experiments on test set. In the initial visualization, ripe in the Ground Truth is represented by dark red, unripe by dark purple-blue, and green by medium gray. After post-processing, ripe is shown in red, unripe in orange, and green in blue. Subfigures **(a–d)** represent four different images, showing the prediction results of the ablation experiments on them.

**Figure 9** presents the qualitative results of the ablation experiments. The results demonstrate that the improved model outperforms the baseline in both occlusion handling and boundary segmentation refinement. In contrast, the original PIDNet tends to miss occluded regions when fruits are partially covered by leaves. Based on SwiftFormer-XS as the backbone, the proposed model effectively captures global contextual information, enabling accurate inference of fruit contours even in occluded regions that are not explicitly annotated in real-world scenarios. Moreover, it alleviates the degradation caused by camera defocus, thereby improving segmentation performance under blurred imaging conditions. This enhancement primarily stems from the integration of the SwiftRep-Hybrid module, which combines the local feature extraction capability of convolution with the global context modeling power of Transformer, thereby improving the model’s ability to perceive complex visual patterns. In addition, the optimized model achieves finer boundary segmentation with clearer and smoother edges, effectively mitigating issues such as boundary blurring and misclassification. This improvement is largely attributed to the Light Fusion Enhance(LFE) module, which incorporates a bidirectional enhancement mechanism and bilinear interpolation to better distinguish foreground objects from background regions. Experimental results demonstrate that the proposed model exhibits strong adaptability to fruit segmentation tasks in complex agricultural environments.

Effectiveness of portfolio losses, As shown in **Table 5**, using only the CE loss achieves a baseline MIoU of 70.90%. Introducing OHEM or boundary loss individually improves performance, indicating that each component contributes to better feature learning. When combining all three loss terms, the model achieves the highest MIoU of 72.20%, demonstrating the effectiveness of the complete loss strategy.

**Table 5:** Ablation study on loss components.

CE	OHEM	Boundary Loss	MIoU (%)
✓	×	×	70.90
✓	✓	×	71.45
✓	×	✓	71.68
✓	✓	✓	**71.20**

Bold represents the best result. MIoU is reported in percentage (%).

### 3.2 Model performance comparison

To further validate the superior performance of the proposed model, four state-of-the-art real-time semantic segmentation models—DeepLab V3+ (Chen et al., 2018), Fast-SCNN (Poudel et al. (2019)), Segformer (Xie et al., 2021), BiSeNetV2 (Yu et al., 2021), STDC (Fan et al., 2021), DDRNet (Hong et al., 2021), and PIDNet-S—were selected for comparison. The evaluation metrics include Mean Intersection over Union (MIoU), Accuracy, Mean Pixel Accuracy (MPA), Macro F1 score, Frames Per Second (FPS), and the number of parameters (Params). These metrics are used to comprehensively assess the performance of the segmentation models.

As shown in **Table 6**, the proposed model achieved outstanding performance compared to state-of-the-art real-time segmentation models. It attained the highest scores in all three accuracy metrics: MIoU (72.20%), Accuracy (99.82%) and MPA (82.48%). Notably, the MIoU surpassed that of the most advanced PIDNet by 2.01%, demonstrating a significant improvement in segmentation precision. In terms of inference speed and model complexity, our model maintained a balanced performance, ranking at a moderate level. Compared to PIDNet, the proposed method not only improved all accuracy metrics but also significantly reduced the number of parameters while increasing inference speed. The model achieved 104.63 FPS, which ensures high real-time responsiveness and meets the requirements for practical deployment. Overall, the comparative results demonstrate that the proposed model exhibits excellent performance in cherry maturity segmentation tasks, effectively balancing segmentation accuracy and efficiency, and fully satisfying the needs of real-time applications.

**Table 6:** Performance comparison of our model and several existing models on the cherry CO Dataset-ripeness val set.

Models	MIoU(%)	Accuracy(%)	MPA(%)	Macro F1(%)	FPS(f/s)	Params(M)
DeepLab V3+ (R50)	66.65	99.80	76.56	80.00	65.65	5.8
Fast-SCNN (CNN)	66.7	99.80	76.21	79.22	**139.38**	**1.4**
Segformer (MiT-B0)	70.55	99.82	80.13	**82.40**	12.28	3.7
BiSeNetV2 (CNN)	50.53	99.68	55.94	61.01	53.18	3.4
STDC (STDC1)	65.30	99.78	74.31	78.12	130.75	8.3
DDRNet (R23-slim)	69.05	99.80	80.26	80.05	110.43	5.7
PIDNet-S (R18)	70.19	99.81	80.04	80.65	100.02	7.6
**Proposed (SwiftFormer-XS)**	**72.20**	**99.82**	**82.48**	81.23	104.63	5.9

Bold represents the best result. Accuracy-related metrics are reported in percentage (%), FPS in frames per second (f/s), and Params in millions (M).

As shown in **Table 7**, the proposed model achieves the highest MIoU (72.20%) while maintaining competitive runtime performance on Jetson TX2 (10.8 FPS, 93 ms latency). Compared to Fast-SCNN and STDC, our method offers significantly better accuracy with only a moderate cost in speed and memory. In contrast, SegFormer performs well in accuracy but is too slow for real-time deployment. These results confirm the suitability of our model for embedded applications.

**Table 7:** Comparison of segmentation performance and runtime efficiency on Jetson TX2 (1312×1312 input, FP16).

Models	MIoU(%)	FPS (f/s)	Memory (MB)	Latency (ms)
DeepLab V3+	66.65	6.8	520	147
Fast-SCNN	66.70	**14.2**	**310**	**70**
Segformer	70.55	1.2	450	833
BiSeNetV2	50.53	5.4	380	185
STDC	65.30	13.5	610	74
DDRNet	69.05	11.2	490	89
PIDNet-S	70.19	10.3	550	97
**Proposed**	**72.20**	10.8	520	93

All models are tested under the same Jetson TX2 settings using TensorRT (FP16) with an input resolution of 1312×1312. Bold indicates the best result in each column. MIoU is reported in percentage (%), FPS in frames per second (f/s), Memory refers to runtime usage (MB), and Latency is measured in milliseconds (ms).

The bold values indicate the best result in each column.

**Table 8** reports per-class IoU to address the dataset’s class imbalance. The proposed model achieves the best results on all three classes, particularly on the underrepresented “green” class. This demonstrates the effectiveness of the combined OHEM and Boundary Loss in improving balanced segmentation performance.

**Table 8:** Per-class IoU comparison on the Cherry CO dataset (Ripe, Unripe, Green).

Models	Ripe	Unripe	Green
DeepLab V3+	64.5%	47.27%	54.96
Fast-SCNN	64.25%	50.62%	52.09%
Segformer	67.58%	55.79%	58.95%
BiSeNetV2	50.41%	20.55%	31.41%
STDC	61.78%	48.5%	51.07%
DDRNet	65.86%	55.19%	55.3%
PIDNet-S	66.87%	57.68%	56.36%
**Proposed**	**67.76%**	**60.81%**	**60.34%**

Bold indicates the best result in each column. IoU is reported in percentage (%).

Considering the motion speed and response latency requirements of cherry-picking robots in orchards (typically above 8 FPS for smooth operation), the 10.8 FPS performance of our model meets the real-time threshold. Therefore, it is feasible for deployment in practical robotic picking scenarios.

**Figure 10:**
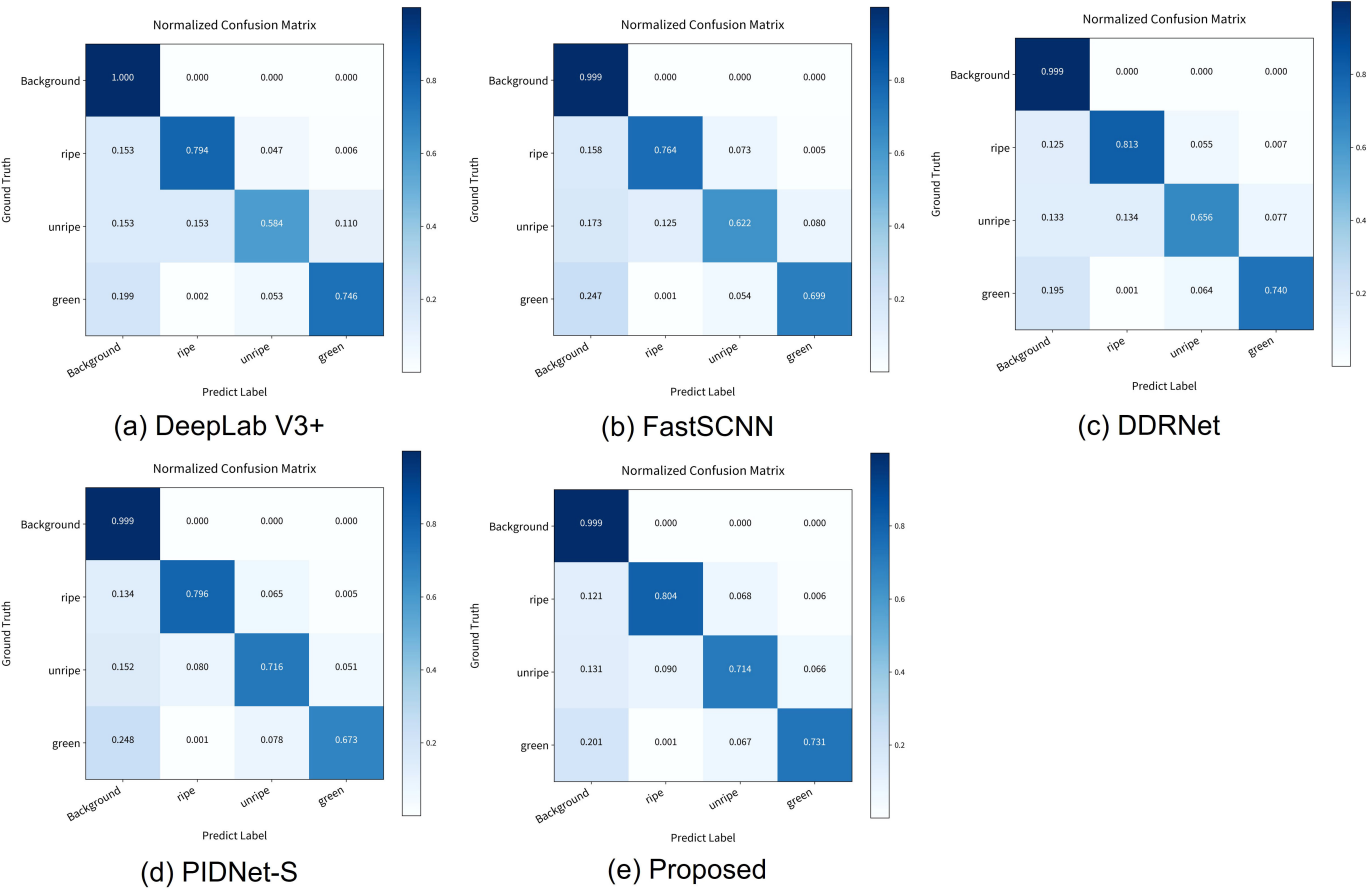
**(a)** DeepLab V3+ normalized per-class prediction accuracy, **(b)** FastSCNN normalized per-class prediction accuracy, **(c)** DDRNet normalized per-class prediction accuracy, **(d)** PIDNet-S normalized per-class prediction accuracy, **(e)** Proposed method normalized per-class prediction accuracy.

Semantic segmentation is essentially a pixel-wise classification task; therefore, confusion matrices are introduced to visualize the maturity recognition capability of different models. The confusion matrices of the five models are shown in **Figure 10**, where the diagonal values represent the pixel-wise accuracy for each class, providing a visual assessment of segmentation performance. The following analysis is based on **Figure 10** and the structural characteristics of each model. We selected the better performing models for confusion matrix analysis. (1) DeepLabV3+ adopts the Atrous Spatial Pyramid Pooling (ASPP) (Chen et al., 2017) module to capture global contextual information using multi-scale dilated convolutions, combined with a lightweight decoder for feature restoration. However, this architecture exhibits certain limitations in the cherry maturity segmentation task, mainly due to its relatively simple decoder design. As a result, under challenging conditions such as lighting variations, object occlusion, and complex backgrounds, severe misclassification occurs between unripe and ripe categories. The classification accuracy for unripe cherries is the lowest, at only 58.4%. (2) Fast-SCNN misclassified 15.8% of ripe pixels as background, 12.5% of unripe pixels as ripe, 17.3% of unripe pixels as background, and 24.7% of green pixels as background. This is mainly because, as a lightweight real-time segmentation network, Fast-SCNN is primarily designed to improve inference speed and omits complex multi-scale feature extraction modules in its architecture. As a result, its segmentation capability becomes insufficient under complex backgrounds, object occlusion, or color similarity. The confusion matrix shows significantly lower diagonal values compared to other models, indicating that Fast-SCNN is not well-suited for cherry maturity segmentation tasks. (3) DDRNet relies on a low-resolution branch to extract semantic information. However, progressive downsampling in this branch leads to class confusion between unripe and ripe cherries, with 13.4% of unripe pixels misclassified as ripe. Compared to the proposed model, the misclassification rate is relatively higher. Moreover, under partial occlusion conditions, the low-resolution features may fail to capture sufficient detail, making it difficult for the model to distinguish between cherries with similar maturity levels. Although DDRNet achieves the highest accuracy for ripe pixels, the classification accuracy for unripe pixels is only 65.6%. This reflects a serious class imbalance problem. (4) PIDNet adopts a three-branch architecture: the P (Proportional) branch captures fine-grained spatial features from high-resolution maps, the I (Integral) branch aggregates local and global contextual information to capture long-range dependencies, and the D (Derivative) branch extracts high-frequency features and predicts boundary regions. However, the model still has room for improvement in high-frequency information extraction and category differentiation. In particular, background misclassification rates remain high for green (24.8%) and ripe (13.4%) classes, indicating that the D branch struggles to accurately distinguish target regions under complex backgrounds. In contrast, the proposed model enhances feature fusion across branches via the LFE module and improves local feature representation through the integration of RepViTBlocks. This effectively reduces misclassification rates, lowering background misclassification for ripe to 12.1% and for green to 20.1%, resulting in overall more stable performance. (5) The confusion matrix of the proposed model shows clearer diagonal color blocks, indicating more stable pixel-level classification performance across different maturity categories. Compared to other models, the proposed model achieves higher pixel-level classification accuracy for all categories. This demonstrates the effectiveness of the proposed method in complex agricultural environments—such as varying lighting conditions, cluttered backgrounds, and fruit occlusion—while alleviating the impact of class imbalance and providing a more reliable visual perception system for cherry-picking robots.

**Figure 11:**
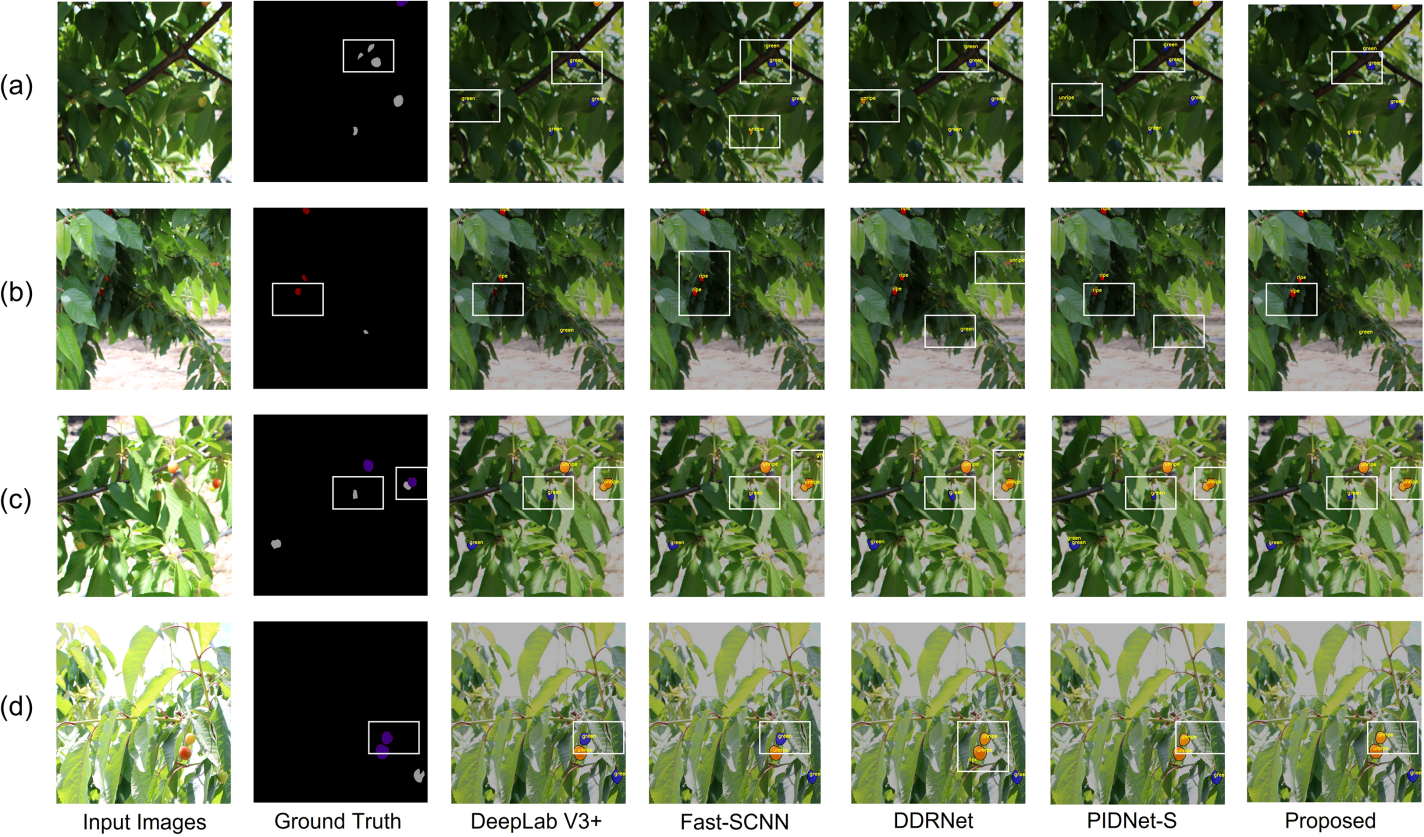
**(a–d)** correspond to four different images, showing the comparison of prediction results by different models.

**Figure 11** illustrates the visualization results of different semantic segmentation models on the test set. We selected the better-performing models for visualization. In the initial visualization, ripe in the Ground Truth is represented by dark red, unripe by dark purple-blue, and green by medium gray. After post-processing, the category colors in the visualization were slightly adjusted to account for variations in outdoor environments. Ripe is shown in red, unripe in orange, and green in blue. White boxes were used to highlight regions of under-segmentation or misclassification. In **Figure 11a**, it can be observed that, except for the proposed model, all other models misclassified some leaves or branches. This misclassification adversely affected segmentation performance. The improved performance can be attributed to the bidirectional enhancement mechanism and bilinear interpolation of the LFE module, which enables the proposed model to accurately identify fruit regions under complex backgrounds, lighting variations, and partial occlusion. This effectively reduces the misclassification of leaves and branches. As shown in **Figure 11b**, DeepLabV3+, FastSCNN, and PIDNet-S failed to identify the corresponding categories. DDRNet exhibited pixel-level misclassification. Only the proposed model accurately recognized all cherry fruits and classified them correctly. This improved performance may be attributed to the combined optimization of global and local features by the LFE module and RepViTBlock. This allows the proposed model to detect and classify all cherry fruits accurately, with no missing categories or misclassification. As shown in **Figure 11c**, Fast-SCNN and DDRNet showed misclassification. DeepLabV3+ and PIDNet-S demonstrated less accurate boundary pixel classification compared to the proposed model. In the rightmost white box, none of the five models successfully distinguished the overlapping unripe and green cherries. This issue may be due to multiple interfering factors, which cause inaccurate segmentation of cherries at different maturity stages. As shown in **Figure 11d**, all models except the proposed one misclassified unripe pixels as green within the white box. PIDNet and other models exhibit limitations in extracting high-frequency boundary information. This results in misclassification of the color transition area between unripe and green cherries. In contrast, RepViTBlock enhances local feature extraction, facilitating more accurate boundary segmentation.

In summary, the proposed method enhances the accuracy of cherry maturity recognition and successfully identifies the maturity level of most cherries. The method effectively reduces the influence of environmental factors and enables richer feature extraction of cherry fruits. It achieves accurate segmentation of cherries at different maturity levels and exhibits strong robustness.

## 4 Conclusion and prospect

This study proposes an optimized real-time semantic segmentation model based on PIDNet for cherry maturity recognition. SwiftFormer-XS was adopted as the backbone network to achieve a balance between segmentation accuracy and computational efficiency. Redundant auxiliary loss functions were removed to significantly reduce the number of model parameters while maintaining high segmentation performance. To further enhance feature extraction, a SwiftRep-Hybrid module was proposed, which combines local feature extraction from convolution with global context modeling from Transformers, enabling the model to capture richer information in complex agricultural environments. In addition, a Light Fusion Enhance (LFE) module was designed, incorporating a bidirectional enhancement mechanism and bilinear interpolation to strengthen feature representations and effectively address challenges such as occlusion, blurriness, and lighting variations. A post-processing module was also introduced to visualize maturity classification results and display precise coordinates of cherries at different maturity levels using a PyQt5-based interface, improving the adaptability of the algorithm for robotic harvesting platforms. Experimental results show that the proposed model achieved an MIoU of over 72.2% and a pixel accuracy (PA) of 99.82%. While maintaining high inference speed and low model complexity, the model outperforms existing real-time segmentation methods such as PIDNet, DDRNet, and Fast-SCNN in terms of segmentation accuracy.

**Figure 12:**
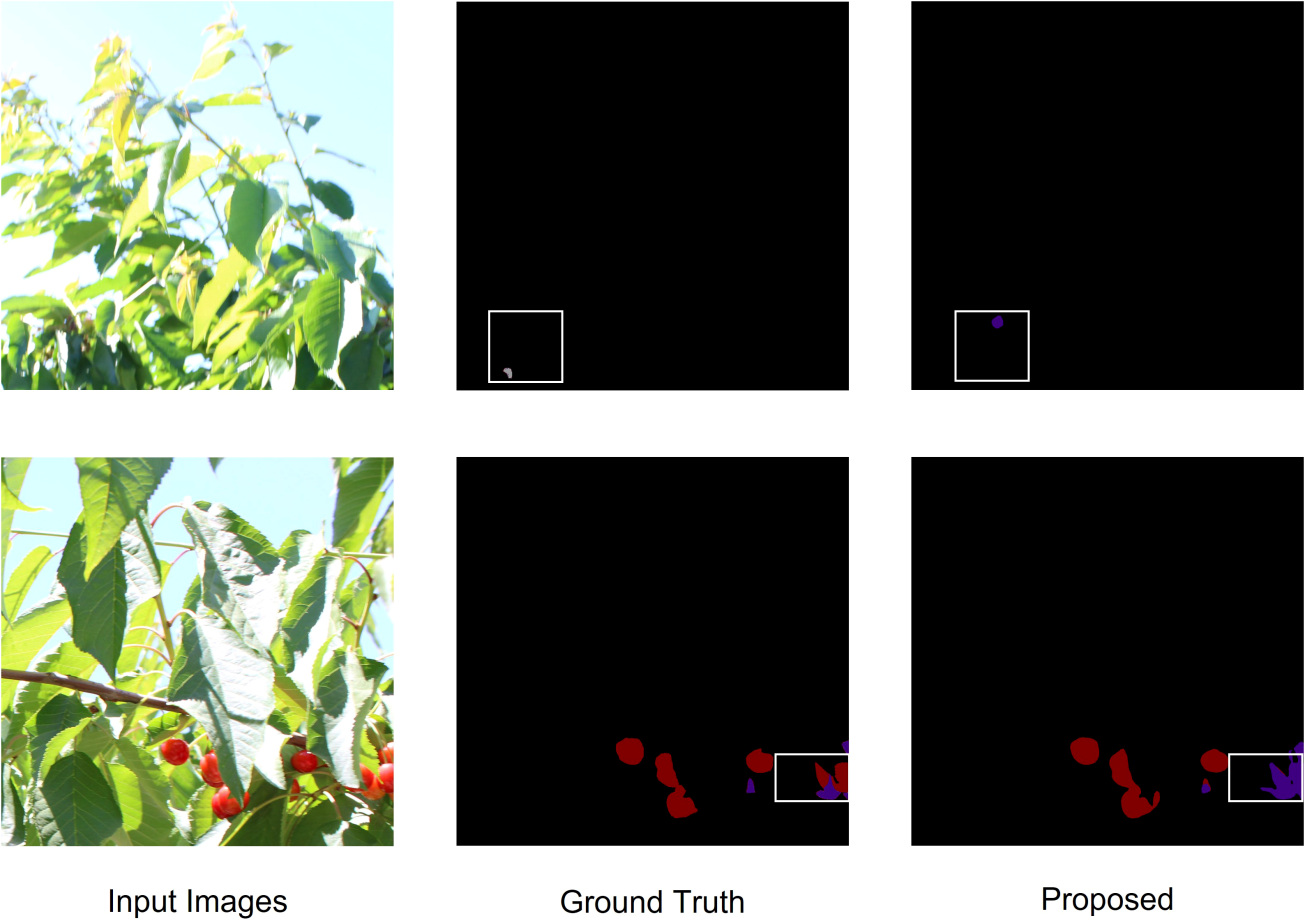
Failure cases of the proposed model.

Although this study has achieved significant progress, several challenges still require further optimization. Firstly, although the model performs well under various lighting conditions, its robustness under extreme environmental factors, such as severe occlusion and highly reflective surfaces, still needs to be improved. **Figure 12** illustrates representative segmentation failure examples, where the model struggles to correctly separate densely clustered fruits or handle intense specular reflections. These cases highlight the limitations of the current approach in real-world complex scenes. In addition, future research could explore multimodal sensor fusion, integrating RGB and depth information to further enhance segmentation accuracy, especially in scenarios with complex backgrounds and occlusion.

In conclusion, this study provides an efficient and accurate real-time semantic segmentation solution for cherry-picking robots. It not only improves segmentation accuracy and computational efficiency, but also enhances adaptability in complex agricultural environments. In the future, by further integrating this method with cherry-picking robots, the proposed method is expected to play an important role in the field of smart agriculture. It offers strong support for the advancement of precision fruit and vegetable harvesting technology.

As a future outlook, the integration of multimodal inputs such as depth or thermal imagery could further enhance robustness and segmentation accuracy, especially under challenging environmental conditions.

## Data Availability

The original contributions presented in the study are included in the article/supplementary material. Further inquiries can be directed to the corresponding author.

## References

[B1] Ali M. Javaid M. Noman M. Fiaz M. Khan S. (2025). “Cosnet: A novel object segmentation network using enhanced boundaries in cluttered scenes,” in Proceedings of the IEEE/CVF Winter Conference on Applications of Computer Vision (WACV) 2024. Piscataway NJ: IEEE.

[B2] Apeinans I. Sondors M. Litavniece L. Kodors S. Zarembo I. Feldmane D. (2024). “Cherry fruitlet detection using yolov5 or yolov8?,” in ENVIRONMENT. TECHNOLOGY. RESOURCES. Proceedings of the 15th International Scientific and Practical Conference. Volume II, Rezekne, Latvia: Rezekne Academy of Technologies. Vol. 2. 29–33.

[B3] Appe S. N. Arulselvi G. Balaji G. (2023). Cam-yolo: tomato detection and classification based on improved yolov5 using combining attention mechanism. PeerJ Comput. Sci. 9, e1463. doi: 10.7717/peerj-cs.1463, PMID: 37547387 PMC10403160

[B4] Bai C. Zhang L. Gao L. Peng L. Li P. Yang L. (2024). Real-time segmentation algorithm of unstructured road scenes based on improved bisenet. J. Real-Time Image Process. 21, 91. doi: 10.1007/s11554-024-01472-2

[B5] Chen L.-C. Papandreou G. Kokkinos I. Murphy K. Yuille A. L. (2017). Deeplab: Semantic image segmentation with deep convolutional nets, atrous convolution, and fully connected crfs. IEEE Trans. Pattern Anal. Mach. Intell. 40, 834–848. doi: 10.1109/TPAMI.2017.2699184, PMID: 28463186

[B6] Chen L.-C. Zhu Y. Papandreou G. Schroff F. Adam H. (2018). “Encoder-decoder with atrous separable convolution for semantic image segmentation,” in Proceedings of the European conference on computer vision (ECCV). Berlin: Springer 801–818.

[B7] Cossio-Montefinale L. Ruiz-del Solar J. Verschae R. (2024). Cherry co dataset: a dataset for cherry detection, segmentation and maturity recognition. IEEE Robotics Automation Lett. 9 6, 5552–5558. doi: 10.1109/LRA.2024.3393214

[B8] Fan M. Lai S. Huang J. Wei X. Chai Z. Luo J.. (2021). “Rethinking bisenet for real-time semantic segmentation,” in Proceedings of the IEEE/CVF Conference on Computer Vision and Pattern Recognition (CVPR) 2021. Piscataway NJ: IEEE. 9716–9725.

[B9] Gai R. Chen N. Yuan H. (2023a). A detection algorithm for cherry fruits based on the improved yolo-v4 model. Neural computing Appl. 35, 13895–13906. doi: 10.1007/s00521-021-06029-z

[B10] Gai R.-L. Wei K. Wang P.-F. (2023b). Ssmda: Self-supervised cherry maturity detection algorithm based on multi-feature contrastive learning. Agriculture 13, 939. doi: 10.3390/agriculture13050939

[B11] Gongal A. Amatya S. Karkee M. Zhang Q. Lewis K. (2015). Sensors and systems for fruit detection and localization: A review. Comput. Electron. Agric. 116, 8–19. doi: 10.1016/j.compag.2015.05.021

[B12] Halstead M. McCool C. Denman S. Perez T. Fookes C. (2018). Fruit quantity and ripeness estimation using a robotic vision system. IEEE robotics automation Lett. 3, 2995–3002. doi: 10.1109/LRA.2018.2849514

[B13] He K. Zhang X. Ren S. Sun J. (2016). “Deep residual learning for image recognition,” in Proceedings of the IEEE Conference on Computer Vision and Pattern Recognition (CVPR) 2016. Piscataway NJ: IEEE 770–778.

[B14] Hong Y. Pan H. Sun W. Jia Y. (2021). Deep dual-resolution networks for real-time and accurate semantic segmentation of road scenes. arXiv preprint arXiv:2101.06085. 24 (3), 3448–3460. doi: 10.1109/TITS.2022.3228042

[B15] Horaud R. Hansard M. Evangelidis G. Ménier C. (2016). An overview of depth cameras and range scanners based on time-of-flight technologies. Mach. Vision Appl. 27, 1005–1020. doi: 10.1007/s00138-016-0784-4

[B16] Huang Z. Wang X. Huang L. Huang C. Wei Y. Liu W. (2019). “Ccnet: Criss-cross attention for semantic segmentation,” in Proceedings of the IEEE/CVF International Conference on Computer Vision (ICCV) 2019. Piscataway NJ: IEEE 603–612., PMID:

[B17] Jing X. Wang Y. Li D. Pan W. (2024). Melon ripeness detection by an improved object detection algorithm for resource constrained environments. Plant Methods 20, 127. doi: 10.1186/s13007-024-01259-3, PMID: 39152496 PMC11328389

[B18] Kang S. Li D. Li B. Zhu J. Long S. Wang J. (2024). Maturity identification and category determination method of broccoli based on semantic segmentation models. Comput. Electron. Agric. 217, 108633. doi: 10.1016/j.compag.2024.108633

[B19] Kervadec H. Bouchtiba J. Desrosiers C. Granger E. Dolz J. Ayed I. B. (2019). “Boundary loss for highly unbalanced segmentation,” in Proceedings of the International Conference on Medical Imaging with Deep Learning (MIDL) 2019. New York, NY: PMLR (Proceedings of Machine Learning Research) 285–296., PMID:

[B20] Kim S.-J. Jeong S. Kim H. Jeong S. Yun G.-Y. Park K. (2022). “Detecting ripeness of strawberry and coordinates of strawberry stalk using deep learning,” in Proceedings of the 2022 Thirteenth International Conference on Ubiquitous and Future Networks (ICUFN). Piscataway, NJ: IEEE 454–458.

[B21] Kodors S. Zarembo I. Lācis G. Litavniece L. Apeināns I. Sondors M.. (2024). Autonomous yield estimation system for small commercial orchards using uav and ai. Drones 8, 734. doi: 10.3390/drones8120734

[B22] Krizhevsky A. Sutskever I. Hinton G. E. (2012). Imagenet classification with deep convolutional neural networks. Adv. Neural Inf. Process. Syst. 25, 84–90. doi: 10.1145/3065386

[B23] Li Z. Jiang X. Shuai L. Zhang B. Yang Y. Mu J. (2022). A real-time detection algorithm for sweet cherry fruit maturity based on yolox in the natural environment. Agronomy 12, 2482. doi: 10.3390/agronomy12102482

[B24] Li H. Li J. Wei H. Liu Z. Zhan Z. Ren Q. (2024). Slim-neck by gsconv: A lightweight-design for real-time detector architectures. J. Real-Time Image Process. 21, 62. doi: 10.1007/s11554-024-01436-6

[B25] Liu S. Xue J. Zhang T. Lv P. Qin H. Zhao T. (2024). Research progress and prospect of key technologies of fruit target recognition for robotic fruit picking. Front. Plant Sci. 15, 1423338. doi: 10.3389/fpls.2024.1423338, PMID: 39711588 PMC11659763

[B26] Liu X. Zhang F. (2021). Extrinsic calibration of multiple lidars of small fov in targetless environments. IEEE Robotics Automation Lett. 6, 2036–2043. doi: 10.1109/LSP.2016.

[B27] Luhmann T. Fraser C. Maas H.-G. (2016). Sensor modelling and camera calibration for close-range photogrammetry. ISPRS J. Photogrammetry Remote Sens. 115, 37–46. doi: 10.1016/j.isprsjprs.2015.10.006

[B28] Maru M. B. Lee D. Tola K. D. Park S. (2020). Comparison of depth camera and terrestrial laser scanner in monitoring structural deflections. Sensors 21, 201. doi: 10.3390/s21010201, PMID: 33396836 PMC7796294

[B29] Ni X. Li C. Jiang H. Takeda F. (2020). Deep learning image segmentation and extraction of blueberry fruit traits associated with harvestability and yield. Horticulture Res. 7. doi: 10.1038/s41438-020-0323-3, PMID: 32637138 PMC7326978

[B30] Poudel R. P. Liwicki S. Cipolla R. (2019). Fast-scnn: Fast semantic segmentation network. arXiv preprint arXiv:1902.04502. doi: 10.48550/arXiv.1902.04502

[B31] Saha K. K. Weltzien C. Bookhagen B. Zude-Sasse M. (2024). Chlorophyll content estimation and ripeness detection in tomato fruit based on ndvi from dual wavelength lidar point cloud data. J. Food Eng. 383, 112218. doi: 10.1016/j.jfoodeng.2024.112218

[B32] Sandler M. Howard A. Zhu M. Zhmoginov A. Chen L.-C. (2018). “Mobilenetv2: Inverted residuals and linear bottlenecks,” in Proceedings of the IEEE Conference on Computer Vision and Pattern Recognition (CVPR) 2018. Piscataway NJ: IEEE. 4510–4520.

[B33] Shaker A. Maaz M. Rasheed H. Khan S. Yang M.-H. Khan F. S. (2023). “Swiftformer: Efficient additive attention for transformer-based real-time mobile vision applications,” in Proceedings of the IEEE/CVF International Conference on Computer Vision (ICCV) 2023. Piscataway NJ: IEEE. 17425–17436.

[B34] Shrivastava A. Gupta A. Girshick R. (2016). “Training region-based object detectors with online hard example mining,” in Proceedings of the IEEE Conference on Computer Vision and Pattern Recognition (CVPR) 2016. Piscataway NJ: IEEE 761–769.

[B35] SIfre L. Mallat S. (2014). Rigid-motion scattering for texture classiflcation. Int. J. Comput. Vision. 2014, 3559:501–515 doi: 10.1007/11503415_34

[B36] Tang Y. Chen M. Wang C. Luo L. Li J. Lian G.. (2020). Recognition and localization methods for vision-based fruit picking robots: A review. Front. Plant Sci. 11, 510. doi: 10.3389/fpls.2020.00510, PMID: 32508853 PMC7250149

[B37] Vaswani A. Shazeer N. Parmar N. Uszkoreit J. Jones L. Gomez A. N.. (2017). Attention is all you need. Adv. Neural Inf. Process. Syst. 30, 6000–6010. doi: 10.5555/3295222.3295349

[B38] Wang A. Chen H. Lin Z. Han J. Ding G. (2024). “Repvit: Revisiting mobile cnn from vit perspective,” in Proceedings of the IEEE Conference on Computer Vision and Pattern Recognition (CVPR) 2024. Piscataway NJ: IEEE. 15909–15920.

[B39] Wang Z. Ling Y. Wang X. Meng D. Nie L. An G.. (2022a). An improved faster r-cnn model for multi-object tomato maturity detection in complex scenarios. Ecol. Inf. 72, 101886. doi: 10.1016/j.ecoinf.2022.101886

[B40] Wang Z. Xun Y. Wang Y. Yang Q. (2022b). Review of smart robots for fruit and vegetable picking in agriculture. Int. J. Agric. Biol. Eng. 15, 33–54. doi: 10.25165/j.ijabe.20221501.7232

[B41] Xie Z. Ke Z. Chen K. Wang Y. Tang Y. Wang W. (2024). A lightweight deep learning semantic segmentation model for optical-image-based post-harvest fruit ripeness analysis of sugar apples (annona squamosa). Agriculture 14, 591. doi: 10.3390/agriculture14040591

[B42] Xie E. Wang W. Yu Z. Anandkumar A. Alvarez J. M. Luo P. (2021). Segformer: Simple and efficient design for semantic segmentation with transformers. Adv. Neural Inf. Process. Syst. 34, 12077–12090. doi: 10.48550/arXiv.2105.15203

[B43] Xu D. Ren R. Zhao H. Zhang S. (2024). Intelligent detection of muskmelon ripeness in greenhouse environment based on yolo-rfew. Agronomy 14, 1091. doi: 10.3390/agronomy14061091

[B44] Xu J. Xiong Z. Bhattacharyya S. P. (2023). “Pidnet: A real-time semantic segmentation network inspired by pid controllers,” in Proceedings of the IEEE Conference on Computer Vision and Pattern Recognition (CVPR) 2023. Piscataway NJ: IEEE 19529–19539.

[B45] Yu C. Gao C. Wang J. Yu G. Shen C. Sang N. (2021). Bisenet v2: Bilateral network with guided aggregation for real-time semantic segmentation. Int. J. Comput. Vision 129, 3051–3068. doi: 10.1007/s11263-021-01515-2

[B46] Zhang J.-q. Wang D. Wei T. Lai X. Tang G. Wang L.-H.. (2024). First report of epicoccum nigrum causing brown leaf spot of sweet cherry (prunus avium) in China. Plant Dis. 108, 2217. doi: 10.1094/PDIS-10-23-2074-PDN

[B47] Zhong H. Wang H. Wu Z. Zhang C. Zheng Y. Tang T. (2021). A survey of lidar and camera fusion enhancement. Proc. Comput. Sci. 183, 579–588. doi: 10.1016/j.procs.2021.02.100

[B48] Zhu X. Chen F. Zheng Y. Chen C. Peng X. (2024). Detection of camellia oleifera fruit maturity in orchards based on modified lightweight yolo. Comput. Electron. Agric. 226, 109471. doi: 10.1016/j.compag.2024.109471

